# Evaluation strategies and uncertainty calculation of isotope amount ratios measured by MC ICP-MS on the example of Sr

**DOI:** 10.1007/s00216-015-9003-9

**Published:** 2015-10-15

**Authors:** Monika Horsky, Johanna Irrgeher, Thomas Prohaska

**Affiliations:** Department of Chemistry, Division of Analytical Chemistry, VIRIS Laboratory, University of Natural Resources and Life Sciences, Vienna, Konrad-Lorenz-Str. 24, 3430 Tulln, Austria; Institute for Coastal Research, Department for Marine Bioanalytical Chemistry, Helmholtz-Centre for Materials and Coastal Research, Max-Planck Straße 1, 21502 Geesthacht, Germany

**Keywords:** Uncertainty of measurement, Isotope amount ratio, Strontium isotopes, MC ICP-MS, Instrumental isotopic fractionation

## Abstract

This paper critically reviews the state-of-the-art of isotope amount ratio measurements by solution-based multi-collector inductively coupled plasma mass spectrometry (MC ICP-MS) and presents guidelines for corresponding data reduction strategies and uncertainty assessments based on the example of *n*(^87^Sr)/*n*(^86^Sr) isotope ratios. This ratio shows variation attributable to natural radiogenic processes and mass-dependent fractionation. The applied calibration strategies can display these differences. In addition, a proper statement of uncertainty of measurement, including all relevant influence quantities, is a metrological prerequisite. A detailed instructive procedure for the calculation of combined uncertainties is presented for Sr isotope amount ratios using three different strategies of correction for instrumental isotopic fractionation (IIF): traditional internal correction, standard-sample bracketing, and a combination of both, using Zr as internal standard. Uncertainties are quantified by means of a Kragten spreadsheet approach, including the consideration of correlations between individual input parameters to the model equation. The resulting uncertainties are compared with uncertainties obtained from the partial derivatives approach and Monte Carlo propagation of distributions. We obtain relative expanded uncertainties (*U*_rel_; *k* = 2) of *n*(^87^Sr)/*n*(^86^Sr) of < 0.03 %, when normalization values are not propagated. A comprehensive propagation, including certified values and the internal normalization ratio in nature, increases relative expanded uncertainties by about factor two and the correction for IIF becomes the major contributor.

**Electronic supplementary material:**

The online version of this article (doi:10.1007/s00216-015-9003-9) contains supplementary material, which is available to authorized users.

## Introduction

The comparability of results of isotope amount ratio measurements depends on the applied calibration strategies and the reported uncertainties. The results of isotope ratio measurements are often indicated with only measurement precision statements on single-sample, repeatability, or reproducibility level instead of expanded measurement uncertainties as recommended by the authoritative Guide to the Expression of Uncertainty in Measurement [1]. In the particular case of Sr isotope ratio measurements, different evaluation strategies are commonly applied, which deliver different extents of information, since the variation of the naturally occurring (‘true’) *n*(^87^Sr)/*n*(^86^Sr) isotope ratio includes radiogenic variation and, to a lesser extent, natural mass-dependent fractionation (MDF). The *n*(^87^Sr)/*n*(^86^Sr) ratio mainly varies according to the radioactive β^-^-decay of ^87^Rb to ^87^Sr, a reaction with a half-life of nearly 50 billion years [2]. As a consequence, the *n*(^87^Sr)/*n*(^86^Sr) ratio is a function of the geological age and the original Rb/Sr ratio [3, 4]; thus the ratio is a fingerprint of its geological source. These properties turned the ^87^Sr/^86^Sr isotope ratio into a highly potential environmental tracer for a remarkable variety of fundamental applications.

Additionally, during the last decade, a variation of the isotope ratio *n*(^88^Sr)/*n*(^86^Sr) has been reported, mainly as a reward for recent advances in mass spectrometry. This ratio had previously been considered invariant in nature, but was found to be affected by mass-dependent isotope fractionation. In fact, the first observation of isotopic fractionation of Sr was reported by Patchett for a meteorite as early as 1980 [5]. It was not until 2006, however, that the study of the variation of *n*(^88^Sr)/*n*(^86^Sr) in nature was introduced to isotope geochemistry [6]. It is usually expressed as a *δ*-value relative to NIST SRM 987. It is evident that natural MDF affects the ratio *n*(^87^Sr)/*n*(^86^Sr), as well. The total range of reported variation to date covers values of *δ*(^88^Sr/^86^Sr) between –1.06(2) and +1.373(7) ‰ [7], whereas the majority of observed values are between 0.1 and 0.5 ‰. Literature references and a detailed evaluation of literature on this issue is given in the Electronic Supplementary Material (ESM, section 2.3.2 and Fig. S3). The above mentioned range of 0.4 ‰ would correspond to about 0.2 ‰ variation of *n*(^87^Sr)/*n*(^86^Sr). This is at least one order of magnitude smaller than the range caused by radiogenic variation. Therefore, MDF effects were hidden or considered irrelevant to the research questions addressed, or it was assumed that the processes controlling isotope fractionation in nature and in the instrument were similar and both could thus be corrected for.

### Correction strategies for instrumental isotopic fractionation (IIF)

IF generally depends on the instrumental setup, ICP conditions, and voltage settings [8–10]. Different approaches to correct for IIF (also termed ‘mass bias’ or ‘mass discrimination’) have been applied when using MC ICP-MS [11]:

- Internal intra-elemental correction (via ^88^Sr/^86^Sr), e.g., [12] – hereafter termed ‘approach 1’
- External intra-elemental correction (standard-sample bracketing) using an isotope certified reference material [13] [in most cases NIST SRM 987 (National Institute of Standards and Technology, Gaithersburg, MD, USA)] e.g., [14] – hereafter termed ‘approach 2’
- Internal inter-elemental correction (e.g., using Zr as internal standard) [15, 16]
- Internal inter-elemental correction using external standards (with Zr as internal standard and standard-sample-bracketing with NIST SRM 987) [17, 18] – hereafter termed ‘approach 3’
- Double spike (DS) approach [19]
- Regression approach [20]

The cited references give examples for application to Sr isotope ratio analyses (except for approach f.). These approaches partly rely on different concepts and are therefore applied in different contexts: The vast majority of studies use approach a. for the calibration of ^87^Sr/^86^Sr data, whereas b. through e. are typically used when other (nonradiogenic) isotope ratios of Sr are (also) of interest.

Different models are applied for internal correction of isotope ratios in MC ICP-MS (strategies a., c., d., and e.): (1) the linear law (which has been shown to be inconsistent in itself [20]), (2) the power law, (3) the exponential law (mathematically equivalent to (2) [21]), (4) Russell’s law [22] – also termed ‘exponential law’ by some authors [20, 23], and (5) the generalized power law [20], of which (2) and (4) represent special cases. The most common model for Sr is the empirical Russell equation. It describes the temporal variation of mass discrimination while the sample is vaporized and thermally ionized in thermal ionization mass spectrometry (TIMS). Variation in instrumental isotopic fractionation in ICP-MS does not follow a systematic temporal pattern, but the same model is applied to account for systematic variation in sensitivity with isotope mass [24]. In addition, IIF is about one order of magnitude larger for MC ICP-MS than for TIMS [23]. The validity of Russell’s law has been disproved for Nd isotope ratios [25, 26]. Paredes et al. found limitations of the model for Sr isotope ratios at very low sample flow rates [27]. For details on the other models, we refer to literature, where the models have been extensively discussed [20, 21, 23, 28], evaluated [29], or expanded [24, 30]. Approach f. is considered a state-of-the-art approach because of its advantage of overcoming limitations of the other internal approaches as it does not strictly rely on a model [29]. Its applicability is, however, limited by the minimal variation in IIF encountered during MC ICP-MS measurements.

In the following study, the focus was laid on approaches a., b., and d. (hereafter termed Approaches 1, 2, and 3), which represent the most common approaches when using a large set of data in e.g., provenance or migration studies. Nonetheless, the same considerations can be assigned to the other concepts.

### Blank correction, interferences, and matrix effects

Not only the Sr amount in the blank affects the final result and its uncertainty, but also its isotopic composition (and how precisely it can be measured). When the main source of ‘background’ Sr is known to be homogeneous regarding its isotopic composition (e.g., from liquid reagents), procedural blanks should not only be used to determine the uncertainty but also to correct the resulting isotope ratio value itself. Often, the source of background Sr is not distinctly known and may be heterogeneous, as it may originate from (minimal) sample carryover during sample preparation procedures, accumulation of Sr from previous samples during the measurement sequence, e.g., in the sample introduction system, insufficient washout, etc.

Efficient analyte/matrix separation is crucial because matrix-based interferences may prevent accurate results [31, 32]. In the case of Sr, isobaric ^87^Rb causes a bias already at trace levels and usually requires mathematical correction. Doubly charged rare earth element interferences may be relevant in silicate rock samples [33], and Ca or P argides or doubly charged Ba argides [34, 35] may be problematic as well. On the other hand, quantitative recovery of Sr is a prerequisite in order to avoid the effect of MDF during separation [36]. Different correction strategies have been described for the correction of isobaric ^86^Kr: by on-peak-zeros only (when bottled high purity Ar with a presumably stable Kr background is used; [37]), or by different mathematical corrections [34, 38, 39]. Moreover, a change in IIF can occur with the introduction of matrix elements into the plasma [9, 24, 40, 41].

### Uncertainty of measurement

The authoritative *Guide to the Expression of Uncertainty in Measurement* (GUM; JCGM 100:2008 [1]; ISO/IEC Guide 98-3:2008) outlines a general procedure for evaluating and expressing uncertainty (of measurement). The EURACHEM/CITAC Guide (QUAM:2012 [42]) specifies an approach for the quantification of uncertainty in analytical chemistry. Both also give detailed introduction into the underlying concepts and the terminology. Measurement uncertainty calculations are carried out using mainly the following approaches: (1) (Gaussian) error propagation (partial derivatives approach) by manual differentiation or as provided by dedicated software packages [e.g., the GUM workbench (Metrodata GmbH, Weil am Rhein, Germany)], (2) the Kragten spreadsheet approach [43], and (3) the ‘propagation of distributions’ approach by Monte Carlo (MC) simulations [44]. (The latter is provided within the GUM workbench software, ver. 2.4, but can also be carried out using spreadsheet software [45].)

Although the majority of publications of (Sr) isotope ratio data do not include statements about measurement uncertainty according to the GUM, several authors have presented uncertainty calculations for Sr isotopes using procedures (1) [17, 18, 46–49], (2) [50–53], and (3) [7, 54, 55]. Examples of uncertainty calculations for other isotope ratios include references [30, 56–62] in which the authors apply (1), references [63–70] in which authors apply (2), and reference [71] in which authors use (3). All approaches use simplifications and can only be considered approximations [72]. (Just as we can never measure a “true” value, but a “best estimate of the true value,” neither can we make a statement of a “true” uncertainty.) Most authors apply a simplified uncertainty calculation based on the assumption that estimates of input quantities are not correlated. The uncertainty of isotope amount ratios can, however, be significantly affected by disregarded correlations [73, 74]. In the GUM workbench software the consideration of correlations can easily be included by entering correlation coefficients into the correlation matrix. In the original Kragten approach, no correlations are considered, but Ellison presented a method to implement correlation correction in a modified spreadsheet approach [75].

A detailed review about isotope ratios measurements by MC ICP-MS gives insight into uncertainty calculations using the partial derivatives approach for different correction strategies for IIF under the assumption of independent input parameters [11]. Uncertainty of Pb isotope ratio measurement by MC ICP-MS, single collector sector field, and quadrupole ICP-MS instruments was compared using the partial derivatives approach, assuming no correlations and using external correction with Tl; contributors including dead-time correction were discussed in detail [63]. Meija and Mester recognized the shortcomings of many uncertainty propagations with respect to the covariance term and therefore investigated the effect of signal correlation on uncertainty propagation in comparison between different ICP-MS instruments [73]. Correlation consideration was consequently applied in a later study for the certification of a reference material [58].

Bürger et al. presented the implementation of the GUM approach for U and Pu isotope ratio measurements using MC TIMS and comprehensively discussed possible uncertainty sources [60, 76]. Uncertainty evaluation for isotope dilution ICP-MS was discussed in detail by many authors, e.g., [77–79]. The importance of correlation consideration has been explicitly recognized in this context [80]. When isotope ratios are measured using counting detectors instead of Faraday cups, additional uncertainty contributions arise from dead time correction [81–83] and correction for secondary electron multiplier nonlinearity [84]. When both detector types are used, yield variation must be accounted for [76]. When very small ratios such as the minor U isotope ratio are measured, peak-tailing effects must be considered as well [85].

Data evaluation in isotope ratio analysis is the way from a measured voltage ratio and basic statistical output from the instrument to a best estimate of the true isotope amount ratio including a realistic uncertainty estimate. In this paper, we compare three commonly used calibration strategies applied to radiogenic Sr isotope ratio data and outline systematic differences between the obtained results depending on the chosen strategy of correction for instrumental isotopic fractionation. Further, we present a full procedure for the calculation of combined uncertainties of [*n*(^87^Sr)/*n*(^86^Sr)] for three selected IIF correction approaches based on a dataset created for wood samples. Complete results of the application study in terms of Sr isotope ratio values will be presented elsewhere.

## Materials and methods

### Sample preparation and measurement

The example dataset used for presenting the different evaluation strategies and uncertainty calculation approaches was generated using the MC ICP-MS instrument Nu Plasma HR (Nu Instruments, Wrexham, UK) with typical instrumental settings given in Table 1.

**Table 1:** MC ICP-MS instrumental and data acquisition parameters (Nu Plasma HR, Nu Instruments, Wrexham, UK)

Instrumental parameter	Value/setting
Plasma power/W	1300
Cool gas flow/L min^–1^	13
Auxiliary gas flow/L min^–1^	0.9
Sampler cone	Ni
Skimmer cone	Ni
Extraction voltage/V	4000
Resolution mode	Low
Sample introduction	DSN-100 with PFA nebulizer
sample uptake rate/μL min^–1^	80
Data acquisition parameter	Value/setting
Scan type	Static
Integration time/s	10
Number of cycles per block	10
Number of blocks	6
Cup configuration (cup: *m/z*)	L5: 83, L4: 84, L3: 85, L2: 86, Ax: 87, H2: 88, H5: 90, H6: 91

Water (18 MΩ cm) obtained from a purification system (ELGA Purelab, ELGA LabWater, High Wycombe, UK) was further purified by sub-boiling distillation (Savillex DST-1000; AHF Analysentechnik, Tübingen, Germany). Concentrated nitric acid (p.a. grade, Merck KGaA, Darmstadt, Germany) was subjected to two sub-boiling distillation steps (Savillex DST-1000) before use.

The samples consisted of wood drill cores that were taken from recent trees in different areas in Austrian forests. Drill cores were air dried, longitudinally cut in halves, and subsequently cut into small splinters. Representative aliquots of ~350 mg wood splinters were digested using microwave assisted acid digestion (Multiwave 3000; Anton Paar, Graz, Austria) using concentrated HNO_3_ and 30 % H_2_O_2_ (Suprapur; Merck KgaA). Sr and other elemental mass fractions were quantified by quadrupole ICP-MS (NexION 300D; Perkin Elmer, Waltham, MA, USA) using standard procedures. Acid digests were evaporated to dryness for preconcentration at 90 °C in PFA vials on a hotplate and redissolved in 8 mol L^–1^ HNO_3_. Samples containing ideally 800–1000 ng Sr were subjected to Sr/matrix separation by Sr specific extraction using Sr Resin with a particle size of 100–150 μm (Triskem, Bruz, France) according to the scheme given in the ESM (Table S1).

Samples were diluted for measurement using dilute nitric acid (*w* = 2 %) to obtain signals of ideally 5–8 V at *m*/*z* 88. The dilute nitric acid was measured as a blank solution prior to every block of four standards and three samples in standard-sample bracketing mode (SSB) and used for further blank correction. The Sr isotopic certified reference material (iCRM) NIST SRM 987 (NIST, Gaithersburg, MD, USA), dissolved in 2 % HNO_3_, was used as a bracketing standard. In order to assess the effect of total Rb/Sr, standard mixtures were prepared from NIST SRM 987 solution and Rb single element standard (Inorganic Ventures, Christiansburg, VA, USA) based on the results from multi-element analysis of separated wood digests by ICP-QMS (NexION 300D): the standard mixtures covered a range of *int*(Rb)/*int*(Sr) between 8.1 • 10^–5^ and 3.5 • 10^–3^ V V^–1^ at the MC ICP-MS instrument. Procedural blanks were processed and measured as samples. Bracketing standards were prepared in a Sr mass fraction range to match that of samples. Diluted Zr standard (Inorganic Ventures, Christiansburg, VA, USA) was added to all standard and sample solutions to obtain similar voltages for ^90^Zr and ^88^Sr. The mass fraction ratio of Zr/Sr in the samples was typically 4–5. In addition, mixtures of Sr (NIST SRM 987) and Zr standards with variable mass fractions were prepared to result in *int*(^90^Zr)/*int*(^88^Sr) between 0.2 and 3.3 V V^–1^ and measured voltages for the two isotopes ^90^Zr and ^88^Sr between 2 and 8 V.

### Data evaluation procedure

Data reduction involves the following corrections: blank correction, correction for interfering ^87^Rb and isotope ratio calibration (a.k.a. ‘correction for instrumental isotopic fractionation (IIF)’ or ‘mass bias correction’). Blank and Rb correction as well as internal intra-elemental IIF correction were implemented in the Nu Instruments Calculation Editor (NICE, Nu Instruments Inc., Wrexham, UK). The NICE routine provides the advantage of data-point-wise correction and automated outlier-elimination separately for each calculation step. Spreadsheet software (Microsoft Excel 2010) was used for compilation of data output and further corrections (standard-sample bracketing). The symbols summarized in Table 2 are used throughout this paper.

**Table 2:** Abbreviations and symbols

*w*	mass fraction
*int*(*i*)	measured voltage at *m*/*z* = *i*
*int*(^*i*^X)	measured voltage corresponding to nuclide ^*i*^X
*n*	amount-of-substance (of an isotope)
*f*_1_	fractionation factor based on *int*(^88^Sr)/*int*(^86^Sr)
*f*_2_	fractionation factor based on *int*(^87^Sr)/*int*(^86^Sr)
*f*_Zr_	fractionation factor based on *int*(^90^Zr)/*int*(^91^Zr)
_cert_	index for ‘certified’ (in NIST SRM 987)
_spl_	index for ‘sample’
_blk_	index for ‘blank solution’
_nat_	index for ‘natural’ (estimated ratio in nature)
_est_	index for ‘estimate’
*M*	nuclide mass / g mol^-1^
avg	average
*r*(*a*,*b*)	correlation coefficient between parameters *a* and *b*
*u*(*a*)	standard uncertainty of *a*
*u*_c_(*a*)	combined uncertainty of *a*
*U*(*a*)	expanded uncertainty of *a*
*k*	coverage factor
*x*_*i*_	input quantity estimate
*U*_*a*_(*b*)	uncertainty contributor of *a* to *U*(*b*)

#### Blank correction

Blank correction was performed via on-peak-zeros (i.e., the subtraction of measured signals at all relevant *m*/*z* in a blank solution from all measured signals in standards and samples). Blank correction is explicitly mentioned in the equations below to allow their use as model equations for subsequent uncertainty calculation. Bottled Ar of 99.999 % purity (Linde Gas GmbH, Stadl-Paura, Austria) was used in the MC ICP-MS. The interference of ^86^Kr was corrected for by blank subtraction. The voltage of ^83^Kr was measured in order to identify Kr background deviations.

#### Correction for interfering ^87^Rb

Even though all samples were matrix separated, the influence of any residual traces of ^87^Rb on the signal at *m*/*z* 87 was corrected for using a simple mathematical correction via peak-stripping: The intensity of ^85^Rb is measured and the contribution of ^87^Rb to the total beam at *m*/*z* 87 is calculated using the *n*(^87^Rb)/*n*(^85^Rb) obtained from the IUPAC/CIAAW tables [86]. As presumably the ‘representative isotopic composition’ was derived from the ‘best measurement of a single terrestrial source’ by rounding to four significant digits, the ratio was calculated from the latter numbers, resulting in a value of 0.38571.

Since Rb is subject to instrumental isotopic fractionation, a correction is required as well. The assumption of equal IIF for Rb and Sr was made in order to correct for the IIF of Rb via the ratio ^88^Sr/^86^Sr (measured in the same sample) applying Russell’s model [22]. (The assumption of equal IIF of different elements has been shown to be incorrect [20, 31]. Nonetheless, we tested the applicability of this approach regarding both the accuracy of the correction and the influence on the combined uncertainty using a series of standard mixtures with increasing Rb content.) This simplified Rb correction approach was used regardless of the subsequent calibration strategy for Sr isotope ratios.

1. Determination of the fractionation factor *f*_1_
  1 $$ {f}_1= \ln \left({\left[\frac{n\left({}^{88}\mathrm{S}\mathrm{r}\right)}{n\left({}^{86}\mathrm{S}\mathrm{r}\right)}\right]}_{\mathrm{cert}}\cdotp {\left(\frac{int{(88)}_{\mathrm{spl}}-int{(88)}_{\mathrm{blk}}}{int{(86)}_{\mathrm{spl}}-int{(86)}_{\mathrm{blk}}}\right)}^{-1}\right)\cdotp {\left( \ln \left(\frac{M\left({}^{88}\mathrm{S}\mathrm{r}\right)}{M\left({}^{86}\mathrm{S}\mathrm{r}\right)}\right)\right)}^{-1} $$
  using [*n*(^88^Sr)/*n*(^86^Sr)]_cert_ = 8.37861.
2. Application of the fractionation factor to Rb
  2 $$ int{\left({}{}^{87}Rb\right)}_{\mathrm{spl}}=\left(int{(85)}_{\mathrm{spl}}-int{(85)}_{\mathrm{blk}}\right)\bullet {\left[\frac{n\left({}{}^{87}\mathrm{R}\mathrm{b}\right)}{n\left({}{}^{85}\mathrm{R}\mathrm{b}\right)}\right]}_{\mathrm{nat}}\bullet {\left(\frac{M\left({}{}^{85}\mathrm{R}\mathrm{b}\right)}{M\left({}{}^{87}\mathrm{R}\mathrm{b}\right)}\right)}^{f_1} $$
  using [*n*(^87^Rb)/*n*(^85^Rb)]_nat_ = 0.38571. The obtained voltage corresponding to ^87^Rb will be subtracted from the total signal at *m*/*z* 87 in the following step.

#### Calibration of isotope ratio measurements

Three different approaches were applied and compared:

1. Internal intra-elemental correction
  In this approach, the ratio *int*(^88^Sr)/*int*(^86^Sr) is measured in the sample and used for correcting the Rb-corrected ratio *int*(^87^Sr)/*int*(^86^Sr) via a theoretical model describing the relation of the extent of instrumental isotopic fractionation to the respective nuclide masses (Eq. 3). *n*(^88^Sr)/*n*(^86^Sr) is subjected to mass-dependent fractionation in nature but unaffected by radiogenic variation. We applied Russell’s exponential model [22]. The fractionation factor *f*_1_ is defined as shown in Eq. 1. In order to account for deviations from Russell’s model observed for the results of the CRM, obtained values were additionally normalized to the mean of four internally corrected *n*(^87^Sr)/*n*(^86^Sr) in NIST SRM 987 solutions measured in the same block (Eq. 3). The certified value [*n*(^87^Sr)/*n*(^86^Sr)]_cert_ = 0.71034 was used as anchor point.
  3 $$ {\left[\frac{n\left({}^{87}\mathrm{S}\mathrm{r}\right)}{n\left({}^{86}\mathrm{S}\mathrm{r}\right)}\right]}_{\mathrm{internal}}=\left(\frac{int{(87)}_{\mathrm{spl}}-int{(87)}_{\mathrm{blk}}-int{\left({}^{87}\mathrm{R}\mathrm{b}\right)}_{\mathrm{spl}}}{int{(86)}_{\mathrm{spl}}-int{(86)}_{\mathrm{blk}}}\right)\cdotp {\left(\frac{M\left({}^{87}\mathrm{S}\mathrm{r}\right)}{M\left({}^{86}\mathrm{S}\mathrm{r}\right)}\right)}^{f_1}\cdotp {\left[\frac{n\left({}^{87}\mathrm{S}\mathrm{r}\right)}{n\left({}^{86}\mathrm{S}\mathrm{r}\right)}\right]}_{\mathrm{cert}}\cdotp {\left(\underset{i=1-4}{\mathrm{avg}}\left({\left[\frac{n\left({}^{87}\mathrm{S}\mathrm{r}\right)}{n\left({}^{86}\mathrm{S}\mathrm{r}\right)}\right]}_{\mathrm{internal},\mathrm{s}\mathrm{t}\mathrm{d}i}\right)\right)}^{-1} $$
2. External intra-elemental correction (standard-sample bracketing)
  The measured ratio *int*(^87^Sr)/*int*(^86^Sr) is corrected via the average of the same ratio measured in certified standard solutions (NIST SRM 987) directly prior (*i* = 1 in Eq. 4) and after each sample (*i* = 2) and its certified value [*n*(^87^Sr)/*n*(^86^Sr)]_cert_ = 0.71034. This correction assumes constant or linearly changing IIF in the course of a measurement sequence, which is not necessarily valid.
  4 $$ {\left[\frac{n\left({}^{87}\mathrm{S}\mathrm{r}\right)}{n\left({}^{86}\mathrm{S}\mathrm{r}\right)}\right]}_{\mathrm{SSB}-\mathrm{S}\mathrm{r}}=\left(\frac{int{(87)}_{\mathrm{spl}}-int{(87)}_{\mathrm{blk}}-int{\left({}^{87}\mathrm{R}\mathrm{b}\right)}_{\mathrm{spl}}}{int{(86)}_{\mathrm{spl}}-int{(86)}_{\mathrm{blk}}}\right)\cdotp \frac{{\left[\frac{n\left({}^{87}\mathrm{S}\mathrm{r}\right)}{n\left({}^{86}\mathrm{S}\mathrm{r}\right)}\right]}_{\mathrm{cert}}}{\underset{i=1,2}{\mathrm{avg}}\left(\frac{int{\left({}^{87}\mathrm{S}\mathrm{r}\right)}_{\mathrm{std}i}}{int{\left({}^{86}\mathrm{S}\mathrm{r}\right)}_{\mathrm{std}i}}\right)} $$
3. Internal inter-elemental correction using external standards (using Zr as internal standard and standard sample-bracketing with NIST SRM 987)
  In this approach, internal correction using another element (Zr), which is added to the Sr solutions, is combined with external correction via bracketing standards. In the first step, the ratio *int*(^87^Sr)/*int*(^86^Sr) in the bracketing standards and the certified value *n*(^87^Sr)/*n*(^86^Sr) are used to determine an estimate of the ‘true’ *n*(^91^Zr)/*n*(^90^Zr) for the Zr standard (which does not necessarily need to be certified for Zr isotope ratios) applying Russell’s model.
  5 $$ {\left[\frac{n\left({}{}^{91}\mathrm{Z}\mathrm{r}\right)}{n\left({}{}^{90}\mathrm{Z}\mathrm{r}\right)}\right]}_{\mathrm{est}}=\underset{i=1,2}{\mathrm{avg}}\left(\left(\frac{int{\left({}{}^{91}\mathrm{Z}\mathrm{r}\right)}_{\mathrm{std}\ i}}{int{\left({}{}^{90}\mathrm{Z}\mathrm{r}\right)}_{\mathrm{std}\ i}}\right)\bullet {\left(\frac{M\left({}{}^{91}\mathrm{Z}\mathrm{r}\right)}{M\left({}{}^{90}\mathrm{Z}\mathrm{r}\right)}\right)}^{f_2}\right) $$
  with
  6 $$ {f}_2= \ln \left({\left[\frac{n\left({}{}^{87}\mathrm{S}\mathrm{r}\right)}{n\left({}{}^{86}\mathrm{S}\mathrm{r}\right)}\right]}_{\mathrm{cert}}\bullet {\left(\frac{int{\left({}{}^{87}\mathrm{S}\mathrm{r}\right)}_{\mathrm{std}\ i}}{int{\left({}{}^{86}\mathrm{S}\mathrm{r}\right)}_{\mathrm{std}\ i}}\right)}^{-1}\right)\bullet {\left( \ln \left(\frac{M\left({}{}^{87}Sr\right)}{M\left({}{}^{86}Sr\right)}\right)\right)}^{-1} $$
  Further, this estimate of *n*(^91^Zr)/*n*(^90^Zr) and the measured *int*(^91^Zr)/*int*(^90^Zr) in the sample are used to determine the fractionation factor *f*_Zr_ and *n*(^87^Sr)/*n*(^86^Sr) in the sample.
  7 $$ {f}_{Zr}= \ln \left({\left[\frac{n\left({}{}^{91}\mathrm{Z}\mathrm{r}\right)}{n\left({}{}^{90}\mathrm{Z}\mathrm{r}\right)}\right]}_{\mathrm{est}}\bullet {\left(\frac{int{(91)}_{\mathrm{spl}}-int{(91)}_{\mathrm{blk}}}{int{(90)}_{\mathrm{spl}}-int{(90)}_{\mathrm{blk}}}\right)}^{-1}\right)\bullet {\left( \ln \left(\frac{M\left({}{}^{91}Zr\right)}{M\left({}{}^{90}Zr\right)}\right)\right)}^{-1} $$
  8 $$ {\left[\frac{n\left({}^{87}\mathrm{S}\mathrm{r}\right)}{n\left({}^{86}\mathrm{S}\mathrm{r}\right)}\right]}_{\mathrm{SSB}-\mathrm{Z}\mathrm{r}}=\left(\frac{int{(87)}_{\mathrm{spl}}-int{(87)}_{\mathrm{blk}}-int{\left({}^{87}\mathrm{R}\mathrm{b}\right)}_{\mathrm{spl}}}{int{(86)}_{\mathrm{spl}}-int{(86)}_{\mathrm{blk}}}\right)\cdotp {\left(\frac{M\left({}^{87}\mathrm{S}\mathrm{r}\right)}{M\left({}^{86}\mathrm{S}\mathrm{r}\right)}\right)}^{f_{Zr}} $$
  Russell’s model is used assuming equal IIF for Sr and Zr. A systematic error in this assumption is, however, compensated by first transferring the fractionation factor from Sr to Zr in the bracketing standard and, secondly, doing the same from Zr to Sr in the samples.

#### Uncertainty calculation

The procedure suggested in the EURACHEM/CITAC guide [42] was followed. The measurand is the isotope amount ratio *n*(^87^Sr)/*n*(^86^Sr) in the purified Sr fraction of digested wood samples calibrated by three different strategies. Depending on the applied strategy, the model equation differs and, as a consequence, different parameters become input quantities. Therefore, we consider three different measurands: [*n*(^87^Sr)/*n*(^86^Sr)]_internal_, [*n*(^87^Sr)/*n*(^86^Sr)]_SSB-Sr_ and [*n*(^87^Sr)/*n*(^86^Sr)]_SSB-Zr_.

**Fig. 1:**
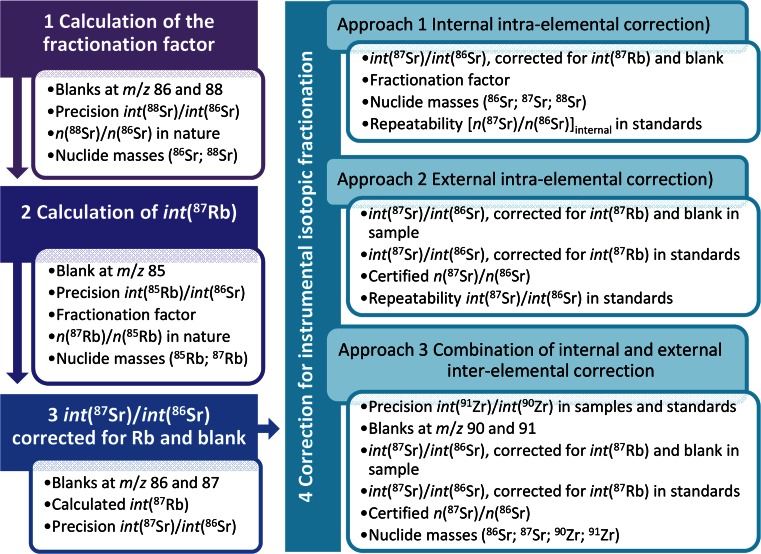
Flowchart specifying the individual data evaluation steps and their associated uncertainty contributors

All estimates of input quantities in Eqs. 1–8 have associated uncertainties. Figure 1 shows a flowchart with all data evaluation steps and the input parameters to the uncertainty calculation. Additionally, correlations between individual input values must be considered. The general equation for uncertainty propagation is given in Eq. 9 in accordance with the GUM [1]. The first term is the well-known expression of the law of propagation of uncertainty for uncorrelated input variables, whereas the second term considers correlations of the input quantities,

9 $$ {u}_{\mathrm{c}}{(y)}^2={\displaystyle {\sum}_{i=1}^n}{\left(\frac{\partial f\left({x}_i\right)}{\partial {x}_i}\bullet u\left({x}_i\right)\right)}^2+2{\displaystyle {\sum}_{i=1}^{n-1}}{\displaystyle {\sum}_{j=i+1}^n}\frac{\partial f\left({x}_i\right)}{\partial {x}_i}\bullet \frac{\partial f\left({x}_j\right)}{\partial {x}_j}\bullet u\left({x}_i\right)\bullet u\left({x}_j\right)\bullet r\left({x}_i,{x}_j\right) $$

where *u*_*c*_(*y*) is the combined uncertainty of the measurand, and *u*_*c*_(*y*)^2^ is the corresponding variance; $\frac{\partial f\left({x}_i\right)}{\partial {x}_i}$ is the partial derivative of the model equation *y* = *f*(*x*_*i*_) for each input quantity estimate *x*_*i*_ (or may also be called sensitivity coefficient); *u*(*x*_*i*_) is the standard uncertainty of an input variable estimate *x*_*i*_ , and *r*(*x*_*i*_, *x*_*j*_) is the correlation coefficient of two input quantity estimates. The product *r*(*x*_*i*_, *x*_*j*_) ∙ *u*(*x*_*i*_) ∙ *u*(*x*_*j*_) is equivalent to the covariance of two input quantity estimates *x*_*i*_ and *x*_*j*_*.* Input quantity estimates will further be referred to as input parameters for ease of reading.

We applied a simplified approach to solve Eq. 9 by means of a Kragten spreadsheet [43]. In this approach, the partial derivatives (differential quotients) are replaced by difference quotients. Standard uncertainties are added to the values of the individual parameters in the diagonal cells of the spreadsheet. For this approach to be valid, the model equations would strictly have to be linear. The errors resulting from the nonlinearity of the model equations were quantitatively assessed by comparing the result from addition of standard uncertainties to each input parameter to the result from subtraction of standard uncertainties. We did not test whether including higher-order terms of the Taylor series expansion (which is the basis of Eq. 9 [1]), would modify the obtained uncertainty for nonlinear equations.

Standard deviations (SDs) should be used as standard uncertainties in the propagation of uncertainties [42]. In case of repeated (independent) observations, SDs of the mean can be used instead. When a confidence interval (CI) is given in literature or on a certificate, the respective value is converted to a SD by dividing by, e.g., 2 in case of a level of confidence of 95 %. When the distribution is unknown and similar probability of all values in the given range is assumed (i.e., rectangular distribution), the stated value is divided by the square root of 3 to obtain the standard uncertainty (SD equivalent) [42].

In order to account for correlations between different input parameters, the classic Kragten approach was extended by an additional line for each measurand to calculate the simplified sensitivity factors $\frac{\varDelta f\left({x}_{\mathrm{i}}\right)}{\varDelta {x}_{\mathrm{i}}}$ and subsequent computation of the correlation term according to Eq. 9. The complete spreadsheet can be found in the ESM. Correlation coefficients (Pearson’s *r*) between input quantities were calculated from raw data using Microsoft Excel. The calculation of the correlation coefficients is sensitive to outliers. Outlier removal generally decreases the SD, while it also reduces the absolute value of the correlation coefficient—in case both parameters were affected by the outlier. When only one of the parameters shows an outlier (e.g., in case of two ratios, or measured voltages corresponding to nuclides of different elements), correlation coefficients may be biased, so outlier-corrected data was used for the calculation of correlation coefficients (and SDs).

In most cases, the correlation term is negative and causes a decrease of the combined uncertainty compared with the simplified approach without the correlation term. In order to be able to present relative contributions of the individual input parameters (grouped for simplicity according the data evaluation steps into measurement precision, blank correction, Rb correction, IIF correction), the squared uncertainty contributors of the relevant (correlated) input quantities were corrected by weighted subtraction of the respective correlation terms.

The uncertainties obtained from the spreadsheet calculations were compared with results obtained by using the GUM Workbench Professional ver. 2.3 (Metrodata GmbH, Weil am Rhein, Germany) with the same model equations and the same input standard uncertainties in order to assess the equivalence of both calculations in spite of the mentioned simplifications in the Kragten approach.

Further, the uncertainty of [*n*(^87^Sr)/*n*(^86^Sr)]_internal_ for one sample was calculated by MC simulation following [45] without consideration of correlations. The result was compared with the result from the Kragten spreadsheet with correlation coefficients set to 0.

#### Uncertainty related to blank correction

While the blank value (measured voltage) used to correct all signals was taken from the on-peak-zero measurement of a blank solution, the influence of sample preparation (procedural blanks) on the combined uncertainty was considered by propagating a respective blank uncertainty. The contribution from Faraday cup baseline variations and Faraday noise were hidden in measured instrumental or procedural blank variations and are not explicitly considered to avoid double counting [76]. Details can be found in the ESM (section 2.1.1).

It is crucial that the blank signal SDs and the correlation coefficient are calculated from the same dataset. A sufficient number of data points is needed to assure a representative correlation coefficient. Therefore, the raw voltage data points from the measurement of a procedural blank (for processed wood samples) or an instrumental blank (for standard solutions measured as samples) were used. Other tested approaches are explained, along with obtained results, in the ESM (section 2.1.2)

Signals measured in blank solutions at the different *m*/*z* of relevance (86, 87, 88) are partly correlated. The signal proportion coming from background Sr has a correlation coefficient close to 1, whereas other components such as electronic noise and contribution of Kr are not correlated, reducing the resulting correlation coefficients.

#### Isotope ratio precision

The quantification of the measurement precision (i.e., voltage ratio precision) is accomplished by calculating the SD during one measurement of a sample. The standard error of the mean of the ratio as calculated by the NICE software was translated to a SD by multiplying by the square root of the number of data points (after outlier elimination). We preferred this approach to the use of standard errors, as we assessed differences in the combined uncertainties related to the different input variables and influence quantities. Therefore, we need uncertainties related to single measurements rather than a method uncertainty like, for instance, a long-term repeatability (standard error of the mean of many independent measurements). Data points obtained from one measurement cannot be considered independent.

Introducing the SD of the individual measured voltages for each *m*/*z* would require the calculation of the covariance between the respective signals. In order to circumvent this, we used the precision of the isotope ratio (SD of the ratio during one measurement) and introduced a precision term to the model equation, which does not change the result value but is associated with an uncertainty. The precision term is an additive term *P*_87/86_ with the value 0 and the SD of the measured ratio (i.e., precision) as its standard uncertainty. It is introduced as shown in Eq. 10. The uncertainties of the measured voltages in the sample are consequently not propagated.

10 $$ y=\dots \left[\frac{int{(87)}_{\mathrm{spl}}-int{(87)}_{\mathrm{blk}}}{int{(86)}_{\mathrm{spl}}-int{(86)}_{\mathrm{blk}}}+{P}_{\frac{87}{86}}\right]\dots $$

#### Uncertainty related to Rb correction

The propagation of the SD of *int*(^85^Rb) would require the calculation of correlation coefficients to all three Sr signals (or ratios) to avoid an overestimation of its influence. Therefore, in analogy to isotope ratio precision consideration, the SD of the ratio *int*(^85^Rb)/*int*(^86^Sr) was calculated and the term for the Rb corrected ratio in Eqs. 3, 4, and 8 is rearranged to facilitate the introduction of both additive precision terms *P*_87/86_ and *P*_85/86_:

11 $$ \left(\frac{int{(87)}_{\mathrm{spl}}-int{(87)}_{\mathrm{blk}}-int{\left({}{}^{87}\mathrm{R}\mathrm{b}\right)}_{\mathrm{spl}}}{int{(86)}_{\mathrm{spl}}-int{(86)}_{\mathrm{blk}}}\right)=\left[\left(\frac{int{(87)}_{\mathrm{spl}}-int{(87)}_{\mathrm{blk}}}{int{(86)}_{\mathrm{spl}}-int{(86)}_{\mathrm{blk}}}\right)+{P}_{\frac{87}{86}}\right]-\left[\left(\frac{int{\left({}{}^{87}\mathrm{R}\mathrm{b}\right)}_{\mathrm{spl}}}{int{(86)}_{\mathrm{spl}}-int{(86)}_{\mathrm{blk}}}\right)+{P}_{\frac{85}{86}}\right] $$

The assessment of the uncertainty of the ratio *n*(^87^Rb)/*n*(^85^Rb) in nature is not trivial. The uncertainties of the abundances as stated by the IUPAC/CIAAW (column “Representative isotopic composition” in [86]) were propagated assuming rectangular distribution and taking into account a correlation coefficient of –1 between the two isotope abundances. The resulting relative standard uncertainty of *n*(^87^Rb)/*n*(^85^Rb) is 0.058 % (normal distribution). This value is larger than literature values for individual measurements or statements of estimated stability of the Rb isotope ratio in nature (e.g., [87, 88]). It should well represent random natural Rb (to the best of the current knowledge since no probability density function is known) and is in accordance with an IUPAC technical report stating maximum variability of *δ(*^87^Rb/^85^Rb) values of 1–2 ‰ [13].

#### Uncertainty of isotope ratio calibration

Depending on the IIF correction approach, different input parameters influence the uncertainty introduced by the correction for IIF (Fig. 1). Uncertainty contributors from uncertainties of nuclide masses [89] were found to be negligible in all cases and will not be further discussed.

All equations contain either certified values of NIST SRM 987 or ‘natural’ isotope ratios. A discussion about which values and associated uncertainties to use is included in the section ‘Calibration strategies’ in ‘Results and discussion’. Uncertainties resulting from propagating these values in comparison to considering them as ‘constants’ are compared.

In approach 1, the measured ratios *int*(^87^Sr)/*int*(^86^Sr) and *int*(^88^Sr)/*int*(^86^Sr) are both input parameters. They share the same denominator and—similar to the signals in the blank solutions—also the measured intensities of the different Sr isotope ratios are supposedly correlated. Correlation coefficients were calculated from blank corrected voltage ratios (*n* = 60 data points) after outlier elimination. The influence of the correlation between the two measured ratios *int*(^87^Sr)/*int*(^86^Sr) and *int*(^88^Sr)/*int*(^86^Sr) on the uncertainty of the ratio [*n*(^87^Sr)/*n*(^86^Sr)]_internal_, was determined. The uncertainty of the normalization step was accounted for by propagating the short-term repeatability of [*n*(^87^Sr)/*n*(^86^Sr)]_internal_ calculated as the SD from four measurements of NIST SRM 987 in one block.

In approach 2, short-term instability in plasma condition may cause serious uncertainties in IIF correction and, thus, may prevent accurate results. Therefore, short time variations in IIF were accounted for by propagating the repeatability expressed in terms of the SD of the *int*(^87^Sr)/*int*(^86^Sr) of four standard measurements (NIST SRM 987) within a block. An additive term *P*_rep_ was introduced into the model equation (Eq. **4**) in analogy to Eq. **10**. Except for the blank signals, there are no further correlated input parameters in this IIF correction approach. Results from approach 3 may be impaired by a variation in the proportion of total Sr to total Zr between bracketing standards and the sample. A possible effect was assessed by analyzing solutions with variable proportions. The influence of a possible correlation between the ratios *int*(^91^Zr)/*int*(^90^Zr) and *int*(^87^Sr)/*int*(^86^Sr) was assessed.

## Results and discussion

The following section is structured with a focus on uncertainty considerations. Individual data evaluation steps are discussed together with the respective associated contributions to the combined uncertainty. The discussion about systematic differences due to different IIF correction strategies is presented in the last part.

### Different uncertainty calculation approaches

An uncertainty calculation for one sample via the partial derivatives approach using the GUM workbench software and the Kragten spreadsheet approach was done to confirm the validity of the set-up Kragten spreadsheet. (During the creation of the spreadsheet, cross checking with the GUM workbench was used to correct or improve the spreadsheet [e.g., it was found that the use of separate Kragten tables for the individual calculation steps (blank correction, Rb-correction, correction for IIF) followed by final combination are not accurate as additional correlations due to shared input parameters are introduced and thus would require separate consideration]. The final resulting uncertainties of both approaches were indistinguishable within three significant digits. The main advantage of the Kragten spreadsheet is its availability. Moreover, transparency and the facility to carry out calculations for a number of samples or standard mixtures with little effort by using references to the output files of the instrument can be seen as an asset.

The difference between the uncertainties for one sample obtained by the MC spreadsheet (mean of 10 uncertainty calculations) and the Kragten spreadsheet approach for internal correction was below 0.1 %. However, the consideration of correlations in the MC approach is not straightforward to implement—at least using a spreadsheet approach [45]. Further, the MC approach does not allow conclusions on the individual contributions from different input parameters. An advantage of the MC approach is the possibility to use input parameters with probability density functions other than normal, rectangular, or triangular. Distributions can be implemented without conversion to standard uncertainties. This could be of interest when probability density functions of isotopic variations [e.g., *n*(^88^Sr)/*n*(^86^Sr) values] in nature are available. The consequence will be resulting uncertainties that cannot be accurately described by an uncertainty value alone (which is by definition normally distributed), but has to include a distribution function.

### Effect of non-linearity of model equations

In the Kragten spreadsheet approach, standard uncertainties are added to the values of the individual parameters. When subtracting individual uncertainties instead of adding them, resulting uncertainties were indistinguishable. The only exceptions are correlated variables. When the change was done for each pair of correlated parameters simultaneously (both uncertainties added versus both subtracted), again no significant alteration of the expanded uncertainty (*k* = 2) within three significant digits was observed.

### Isotope ratio precision

**Fig. 2:**
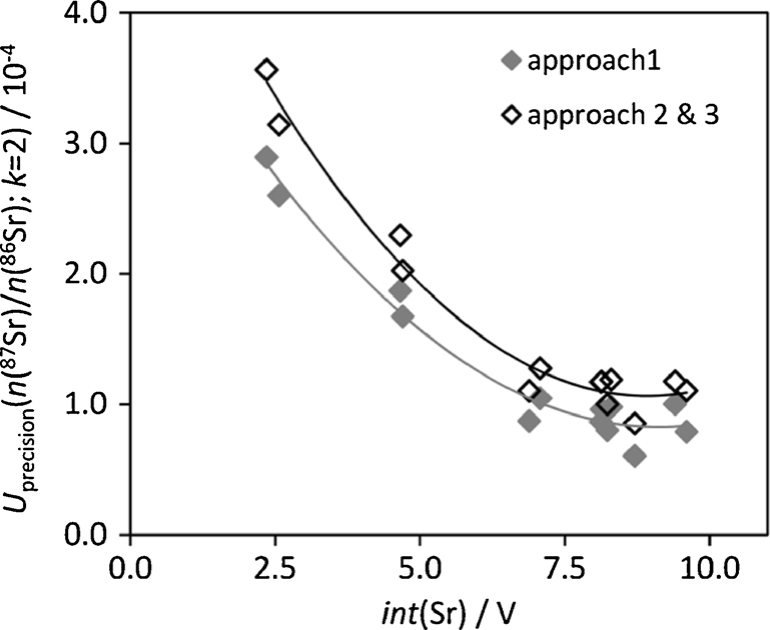
Uncertainty contributor of precision to *U* (*k* = 2) of *n*(^87^Sr)/*n*(^86^Sr) for different IIF correction approaches calculated for a series of standards, related to the total measured voltage for Sr with polynomial trend line

The uncertainty contributor of the isotope ratio precision increases linearly with the relative isotope ratio measurement precision expressed as RSD of *int*(^87^Sr)/*int*(^86^Sr). The precision is inversely related to the total measured voltage and, consequently, so is the uncertainty contributor (Fig. 2). It is evident that the uncertainty contributor can be decreased by measuring at sufficiently high concentrations, with improved sample introduction or by enhancing the sensitivity of the instrument.

The difference between IIF correction approach 1 and the two SSB approaches 2 and 3 (Fig. 2) relates to the consideration of the correlation between the ratios *int*(^88^Sr)/*int*(^86^Sr) and *int*(^87^Sr)/*int*(^86^Sr) for internal correction, which reduces the relative contribution of the isotope ratio precision. (Further details can be found below in section ‘Approach 1’.)

### Blank correction and associated uncertainty

Only the previous on-peak-zeros were used to correct the Sr isotope ratio value for a blank (instrumental background). A possible bias introduced by a procedural blank should be reflected in the uncertainty calculation since the source of background Sr may be heterogeneous.

The uncertainty contributor of the blank correction at *m*/*z* 86, 87, and 88 consists of an uncorrelated term as obtained from Kragten spreadsheet calculations and a term considering the correlation between all three blank signals. In case of sample-standard bracketing, only the correlation between the signals at *m*/*z* 86 and 87 was of relevance as the beam at *m*/*z* 88 only occurs in the Rb correction and the respective correlation terms were negligible. The same is true for the correlation between the blank signal at *m*/*z* 85 and all Sr blank signals—its effect was tested but considered insignificant and therefore omitted in further calculations. When propagating the same blank correction for both the measured *int*(^87^Sr)/*int*(^86^Sr) in the sample and the bracketing standards (approaches 2 and 3), the contribution of the blank correction becomes negligible. Therefore, it was only propagated for the sample (or standard mixture measured as a sample).

Blank SDs and correlation coefficients were calculated from single data points (*n* ~ 60) of the measurement of a procedural blank (for processed wood samples) or an instrumental blank (standard mixture measured as a sample). Considerations about other approaches that were tested can be found in the ESM (section 2.1.2, Fig. S1). Correlation coefficients involving signals at *m*/*z* 86 are usually slightly smaller compared with *r*(*int*(87)_blk_,*int*(88)_blk_), which may relate to the contribution of ^86^Kr. This effect is more pronounced when instrumental blanks are used. Our blank uncertainty contribution accounts for uncertainty caused by variation of ^86^Kr during the measurements. Details on the results of monitoring of ^83^Kr during measurements can be found in the ESM (section 2.1.3).

**Fig. 3:**
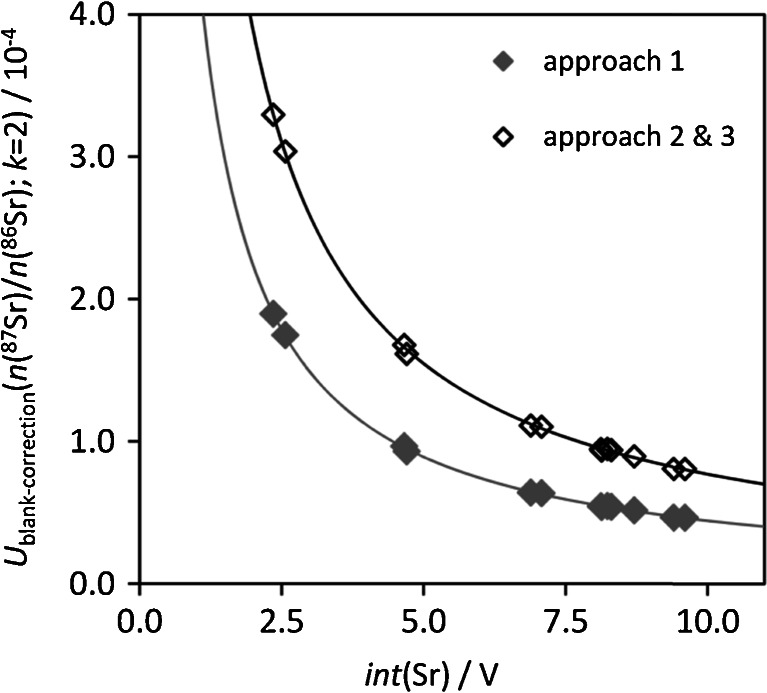
Uncertainty contribution (*k* = 2) of blank correction to the uncertainty of *n*(^87^Sr)/*n*(^86^Sr), determined using standards with variable Sr concentration and one instrumental blank for the different IIF correction approaches

Figure 3 shows the dependence of the uncertainty contributor of the blank correction to the uncertainty of *n*(^87^Sr)/*n*(^86^Sr)—for a series of standards with variable Sr concentration using different IIF correction approaches. The difference between IIF correction approach 1 and approaches 2 and 3 (which give identical blank contributions) relates back to the model equations and particularly to the impact of the correlation correction. The results highlight the importance of measuring at adequate Sr concentrations in the samples to obtain (in the case of the used setup) total Sr signals of >6 V to keep blank uncertainty contributions low.

We tested the shift of *n*(^87^Sr)/*n*(^86^Sr) in wood digests caused by two identically processed procedural blanks. The bias was +0.003 % and –0.004 %, respectively, for IIF correction approach 1, and +0.001 % and +0.004 %, respectively, for approaches 2 and 3. The relative expanded uncertainty introduced by blank correction was 0.005 %. Therefore, the approach was shown to be appropriate since the bias introduced by procedural blanks was sufficiently covered by this uncertainty.

### Correction for interfering ^87^Rb and associated uncertainty

The measured voltage ratio of total Rb to total Sr in Sr and Zr standard mixtures without addition of Rb was between 2 • 10^–5^ and 3 • 10^–4^ V V^–1^ and increased in the prepared standard mixtures with increasing Zr/Sr. This indicates that the Zr standard (1000 mg L^–1^, 99.9944 % purity of the starting material) introduces a Rb background in the low pg range. This points to one disadvantage of the IIF correction approach involving the addition of Zr, while it highlights the importance to carry out a correction for interfering Rb at *m*/*z* 87 for all samples and standards. The range of residual Rb/Sr (total voltage ratio) in samples subjected to Sr/matrix separation is shown in Fig. S2 (ESM).

**Fig. 4:**
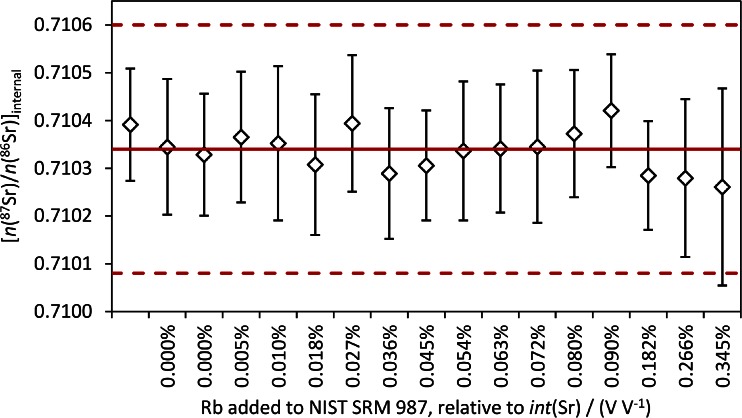
*n*(^87^Sr)/*n*(^86^Sr), corrected for IIF by approach 1, for Sr-Rb standard mixtures with increasing Rb/Sr. Uncertainty bars reflect *U*(*k* = 2). The red solid line corresponds to the certified value of *n*(^87^Sr)/*n*(^86^Sr) in NIST SRM 987, the red dotted lines to the limits of its certified range (95 % CI)

The results for *n*(^87^Sr)/*n*(^86^Sr)_internal_ from measurement of Sr and Rb standard mixtures are shown in Fig. 4. There is no drift with increasing *n*(Rb)/*n*(Sr) and slight variations are within the expanded uncertainty (*k* = 2). In contrast to a previous study where accuracy was compromised at *int*(^85^Rb)/*int*(^88^Sr) levels of 0.005 % [18], we could not observe any effect of the Rb correction on the accuracy of *n*(^87^Sr)/*n*(^86^Sr) within the studied concentration range. Consequently, we did not introduce further terms reflecting, e.g., different IIF for Rb and Sr, into the uncertainty calculation related to the Rb correction.

**Fig. 5:**
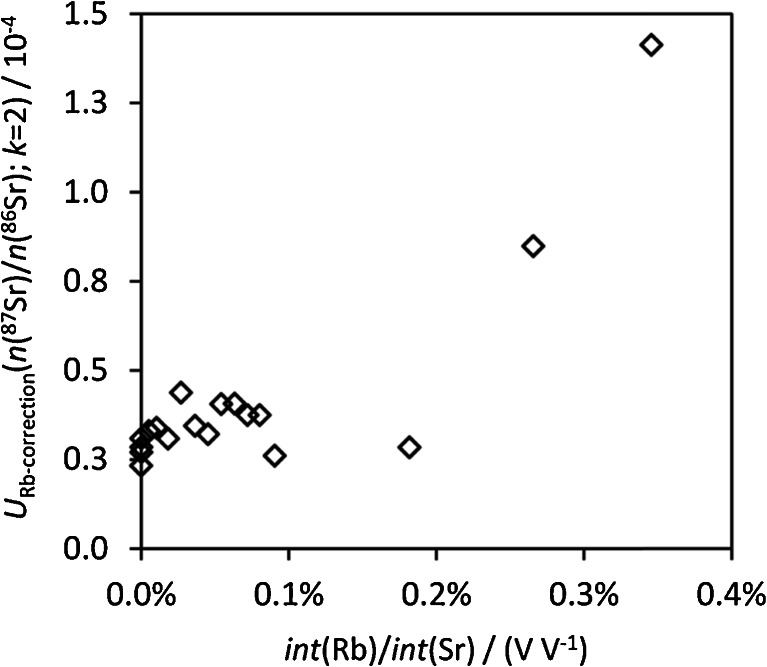
Expanded uncertainty contributor (*k* = 2) of the correction for residual Rb to the uncertainty of *n*(^87^Sr)/*n*(^86^Sr) versus the Rb/Sr voltage ratio measured in standards. There is no significant difference depending on the IIF correction approach

Figure 5 shows the dependence of the uncertainty contribution of Rb correction on the ratio Rb/Sr expressed as measured total voltage ratio. It shows no trend for *int*(Rb)/*int*(Sr) < 0.18 % and increases approximately linearly at higher ratios. The uncertainty contributor consists of the terms related to the precision of *int*(Rb)/*int*(Sr), the natural Rb isotope ratio, and the recorded blank intensity at *m*/*z* = 85. (Strictly, the fractionation factor, which is used in the calculation of the voltage attributable to ^87^Rb, contributes to the uncertainty of the Rb correction. In approach 2, the only contribution of the uncertainty of the fractionation factor relates to the Rb correction. It accounted for maximum 1 % of the total variance contributor related to Rb correction.) The combined Rb correction uncertainty as hypothetical only source of uncertainty of the final isotope ratio would give a relative expanded uncertainty (*k* = 2) of ~0.005 %. When considering all other uncertainty contributors, it relatively accounts for between <1 and 5 % of the variance of *n*(^87^Sr)/*n*(^86^Sr) for ‘typical’ samples with *int*(Rb)/*int*(Sr) up to 0.18 % (depending on the IIF correction strategy and whether certified values and natural ranges are propagated or not).

The most relevant influence parameters to the Rb correction uncertainty contributor are the blank at *m*/*z* 85 and the measurement precision of *int*(^85^Rb)/*int*(^86^Sr). At higher *int*(Rb)/*int*(Sr)—in the tested range of values between 0.18 and 0.35 %—the latter becomes the major contributor. Further details on individual contributors can be found in the ESM (section 2.2.1)

### Calibration strategies

#### Approach 1

Internal intra-elemental correction accounts for both long-term and short-term variation in IIF because the calibrant ratio and the calibrated ratio are measured simultaneously. A possible bias due to different detector efficiency factors [90] is accounted for by final normalization to the block mean value of likewise corrected NIST SRM 987 results using the certified value as anchor point.

The major concern in this correction approach is the value that is taken as *n*(^88^Sr)/*n*(^86^Sr) in internal intra-elemental normalization [91]. Traditionally, the value 8.375209 (or its reciprocal 0.1194, as published by Nier in 1938 [92]) is being used. Details about the implementation of this value and its alternatives are described in the ESM (section 2.3.1, Table S2) The certificate of NIST SRM 987 gives a value of 8.37861. When the latter is used to calculate [*n*(^87^Sr)/*n*(^86^Sr)]_internal_ of NIST SRM 987, the result compares with the value on the certificate (0.71034). When, however, Nier’s value is used, the certificate value for *n*(^87^Sr)/*n*(^86^Sr) is not valid and, instead, a ‘corrected’ value of 0.7101938 [93] should be used as a reference to compare with, which is, however, different from several so-called ‘accepted’ values from literature, typically determined by long-term TIMS. They range from 0.71024 [15] and 0.710245 [16] to 0.710263 [94]. One reason why these values are preferred to the certified value is their smaller uncertainty [16].

When dealing with random ‘real world’ material, the accuracy of using the mentioned *n*(^88^Sr)/*n*(^86^Sr) value from the NIST SRM 987 certificate is questionable. Several studies investigating *δ*(^88^Sr/^86^Sr) have found on the one hand a variation and on the other hand significantly different average values for e.g., the bulk silicate earth (0.27(5) ‰; 2 SD, *n* = 8) [38] or seawater (0.386(5) ‰ (2 *SEM*, *n* = 10), OSIL IAPSO seawater standard [95]). Forty-one water samples from rivers from four continents and volcanic islands were investigated by DS TIMS and yielded an average value of 0.32(17) ‰ (2 SD, *n* = 41) [96]. Measurement results from certified reference materials regarding *δ*(^88^Sr/^86^Sr)_SRM_987_ were compiled by Brand et al. and cover values between –0.20(2) and +0.54(3) ‰ [13]. The mentioned datasets give an indication that the ‘representative isotopic composition’ of real world samples would probably have an average shifted to higher *n*(^88^Sr)/*n*(^86^Sr) compared with NIST SRM 987, at least when considering rock, soil, or water samples. A short review of publications giving *δ*(^88^Sr/^86^Sr) is included in the ESM (section 2.3.2, Fig. S3). This further supports the approach not to use Nier’s value, which would correspond to a *δ*(^88^Sr/^86^Sr)_SRM_987,certificate_ of –0.4 ‰. A summary of possible values for *n*(^88^Sr)/*n*(^86^Sr) with arguments in favor of and against their utilization for internal normalization is included in the ESM (Table S2). We used the value 8.37861 from the NIST SRM 987 certificate to retain the validity of the certificate and in order to apply the same value to standards and samples.

The correlation coefficient between the two measured ratios *int*(^87^Sr)/*int*(^86^Sr) and *int*(^88^Sr)/*int*(^86^Sr) was determined for seven samples from outlier-corrected raw data and resulted in values between 0.40 and 0.60. A variation of the correlation coefficients in this range causes relative differences in the combined uncertainty of 3–5 % (*n* = 8). Therefore, a correlation coefficient of 0.5 was used as an appropriate estimate for all further calculations, which is the expected value for ratios close to unity [23]. The combined uncertainty was decreased by 15(4)% (2 SD, *n* = 8) when considering the stated correlation in contrast to ignoring it—this decrease can be assigned both to the precision of *int*(^87^Sr)/*int*(^86^Sr) and the IIF correction term.

#### Approach 2

Standard-sample bracketing using the same ratio in samples and standards is a very straightforward methodology and does not require any theoretical model or assumptions about certain isotope ratios in nature. Additionally, possible differences in Faraday cup efficiencies and gains do not bias the results because the same set of detectors is used for both the standard and the sample [90]. When absolute values are reported, the reference value of the bracketing standard must be clearly stated. We used NIST SRM 987 and the value from the certificate. Uncertainty calculation is consequently equally uncomplicated and does not require further correlation terms. Another advantage of SSB-Sr is the direct determination of delta values (potentially for all Sr isotope ratios), which do not rely on any certified values and their uncertainties (except from inhomogeneities of the reference material).

Long-term time-dependent fluctuations of IIF (between measurements) are corrected for by averaging measured ratios between a standard prior to and after the sample, whereas changes at shorter frequencies (within measurements) may bias the results. This can be accounted for in the uncertainty calculation by propagating short-term repeatability, obtained from measurement of NIST SRM 987. Possible mass-dependent fractionation occurring during Sr/matrix separation (e.g., when recoveries are incomplete) are not corrected for either (these are accounted for only in approach 1). It is therefore recommended to monitor *n*(^88^Sr)/*n*(^86^Sr) as well as elemental contents after Sr/matrix separation along with Sr recovery. Ideally, accuracy of this approach should be controlled using matrix iCRMs. (No such material is available to date for wood to the best of the authors’ knowledge).

#### Approach 3

This approach assumes equal instrumental isotopic fractionation for the sample solution and the standard—a prerequisite that is approached by near perfect matrix matching (concentration matching and removal of matrix components) in the sample solution. Both short- and long-term fluctuations in IIF are corrected for. Any mass-dependent fractionation occurring prior to measurement is, however, not accounted for (see above).

**Fig. 6:**
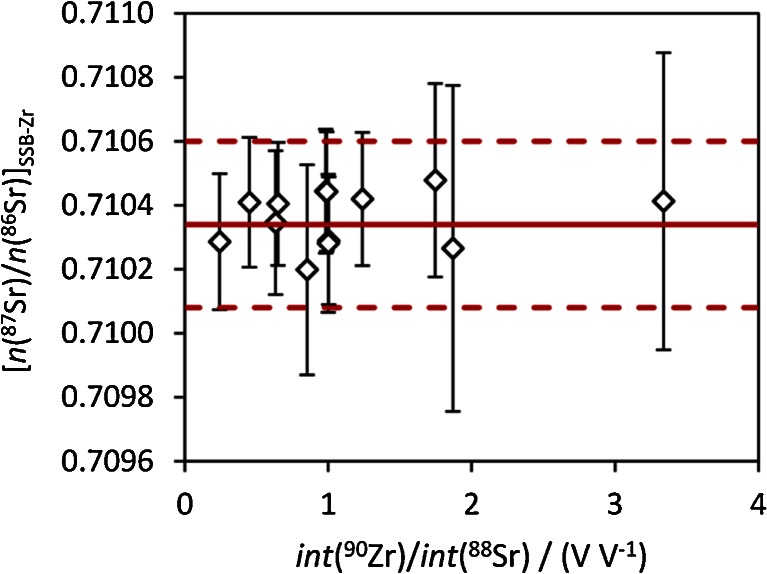
*n*(^87^Sr)/*n*(^86^Sr) corrected by IIF correction approach 3 (SSB-Zr) for a series of standard with varying Zr/Sr ratio given as voltage ratio of both most abundant isotopes. Error bars correspond to expanded uncertainties (*k* = 2) without propagation of the uncertainty of the certified value. Measured voltages for both isotopes range from 2 to 8 V. The red solid line corresponds to the certified value of *n*(^87^Sr)/*n*(^86^Sr) in NIST SRM 987, the red dotted lines to the limits of its certified range (95 % CI)

Variation of IIF between samples and standards might be caused by insufficient concentration matching of Sr and/or Zr and, consequently, differing Zr/Sr ratios. This was tested using standard mixtures. We could not find any systematic bias caused by variations of both elemental concentrations corresponding to voltages between 2 and 8 V for the isotopes ^90^Zr and ^88^Sr and voltage ratios between 0.24 and 3.3 (Fig. 6). In contrast, Yang et al. observed an effect of the Zr and Sr concentration on *n*(^88^Sr)/*n*(^86^Sr) in standard mixtures (corrected using the same ratio in bracketing standards) and highlighted the importance of close concentration matching of the internal standard [17]. We assent to this view because possible effects may differ, for instance, depending on instrumental parameters (including sample introduction).

In addition to the SD of the measured *int*(^87^Sr)/*int*(^86^Sr) in sample and bracketing standards, the SD of the three measured ratios *int*(^91^Zr)/*int*(^90^Zr) add contributions to the combined uncertainty, which increase linearly with the respective precisions; an example is shown in the ESM (Fig. S4). When comparing repeatability of the different approaches, however, Yang et al. found an improvement for the combination of SSB and internal normalization using Zr [17]. They report expanded uncertainties (*k* = 2) for the isotope abundances of ^86^Sr, ^87^Sr, and ^88^Sr in a reference material of 0.047, 0.021, and 0.010 %, respectively [17].

The correlation between the measured ratios *int*(^91^Zr)/*int*(^90^Zr) and *int*(^87^Sr)/*int*(^86^Sr) was dependent on the daily tuning. Values of *r* were usually in the range between –0.3 and +0.3 (*n* = 60 data points), which correspond to *P* values of ≥0.03. A substantial proportion of samples therefore showed insignificant correlation when assigning a significance level of 0.05. Since both positive and negative correlations occurred, a possible correlation between the *int*(^91^Zr)/*int*(^90^Zr) and *int*(^87^Sr)/*int*(^86^Sr) was not considered in further calculations. The lack of correlation between the two ratios indicates that our data do not reflect the basic assumption of approach 3 that any short-term variation in IIF would affect both the Zr and Sr isotope ratio similarly. This is probably due to a lack of these IIF variations and may suggest that approach 3 does not provide advantages compared with approach 2 for our setup.

#### Comparison of uncertainty contributors for the 3 approaches

**Fig. 7:**
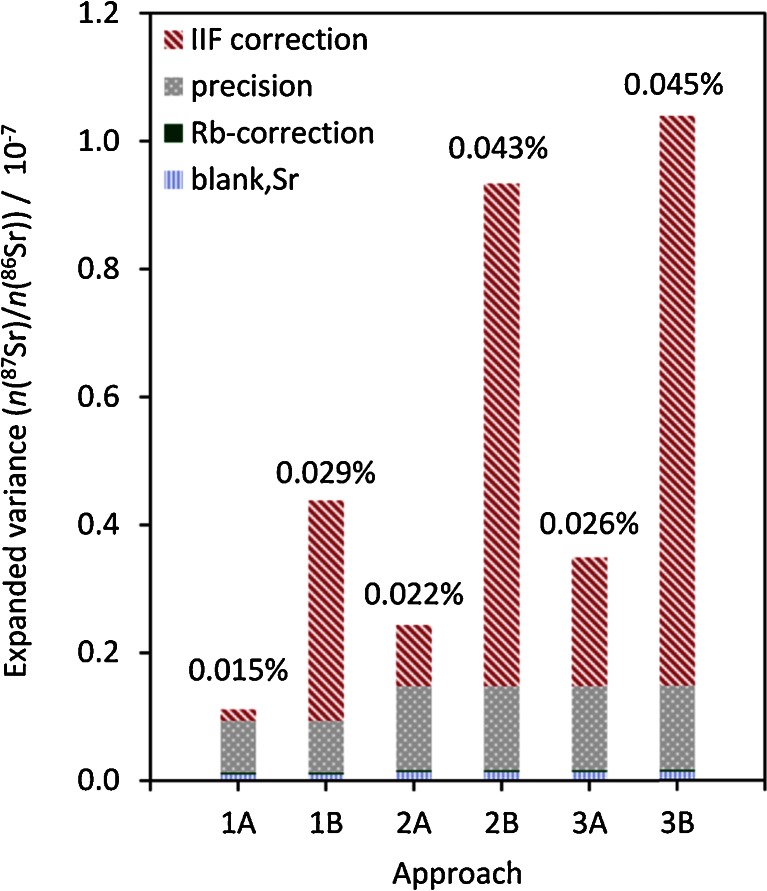
Absolute contributions of corrections to the expanded variance of *n*(^87^Sr)/*n*(^86^Sr) [= *U* ^*2*^ (*k*(*U*) = 2)] for one wood digest sample using different IIF correction approaches (1, 2, 3); (**A**) without, (**B**) with propagation of uncertainty of estimated natural range (1B) or certified values (2B, 3B). Numbers on top of bars show corresponding relative expanded uncertainties *U*_rel_ (*k* = 2)

Figure 7 shows the uncertainty contributions of measurement precision and all data evaluation steps in the dimension of expanded variance (squared expanded uncertainty) that sum up to the expanded variance of the Sr isotope ratio of interest, for the three different IIF correction approaches in comparison. When uncertainties of ‘normalization values’ [e.g., certified values of NIST SRM 987 or estimated natural range of *n*(^88^Sr)/*n*(^86^Sr)] are not propagated (cases 1A, 2A, 3A in Fig. 7), the resulting relative expanded uncertainties *U*_rel_ (*k* = 2) are <0.03 % and the measurement precision is a major contributor.

When the uncertainties of the ‘normalization values’ are propagated, it is evident that the major contributor to the uncertainty in all three approaches is the uncertainty of the certified value or of the ‘natural’ internal normalization ratio (cases 1B, 2B, 3B in Fig. 7). This is in accordance with previous results [17]. The uncertainty of the certified value of NIST SRM 987 used as anchor point for normalization in approach 1 was not propagated. (It would shift the relative expanded uncertainty for case 1B to 0.048 %).

### Comparison to published uncertainties

Our uncertainty values agree well with those of Fortunato et al. who determined a relative combined uncertainty of 0.016 % for internal normalization of *n*(^87^Sr)/*n*(^86^Sr) with the major contribution arising from correction for IIF including the certificate-stated uncertainty of *n*(^88^Sr)/*n*(^86^Sr) [46]. In a study of mineral waters, Brach-Papa et al. present uncertainty budgets for different scenarios with relative contributions and end up with relative expanded uncertainties between 0.004 and 0.03 % [47]. The latter value refers to the most comprehensive propagation, where the major contributor is, again, the uncertainty related to internal IIF correction, where they propagate the difference in the final ratio between two possible normalization values [47]. (They propagate relative shifts of a result value as standard uncertainty of a unity factor). The approach was also adopted by Paredes et al. who worked at very low sample flow rates and reported expanded uncertainties (*k* = 2) between 0.007 and 0.07 % when propagating only repeatability, blank correction, and a component accounting for the difference between the observed and the certified *n*(^87^Sr)/*n*(^86^Sr) [49].

In contrast, Garcia-Ruiz et al. determined a relative combined uncertainty (*u*_c,rel_) of 0.052 % for the validation of on-line Sr/Rb separation for ciders [50]. They found that the blank correction is the major uncertainty contributor (42 %), followed by measurement precision (38 %) and Rb (blank) correction (12 %). The contribution of internal IIF correction was considered insignificant (8 %). Irrgeher et al*.* applied the partial derivatives approach and report a relative expanded uncertainty (*U*_rel_; *k* = 2) of 0.014 % for *n*(^87^Sr)/*n*(^86^Sr) in a biological reference material, but do not include details about the individual contributors [18]. A value of 0.014 % was also reported by Rodríguez-Castrillón et al. for *u*_c,rel_ for a methodology combining on-line chemical separation with multiple linear regression for data evaluation [51].

### Comparison of obtained results for different calibration strategies

**Fig. 8:**
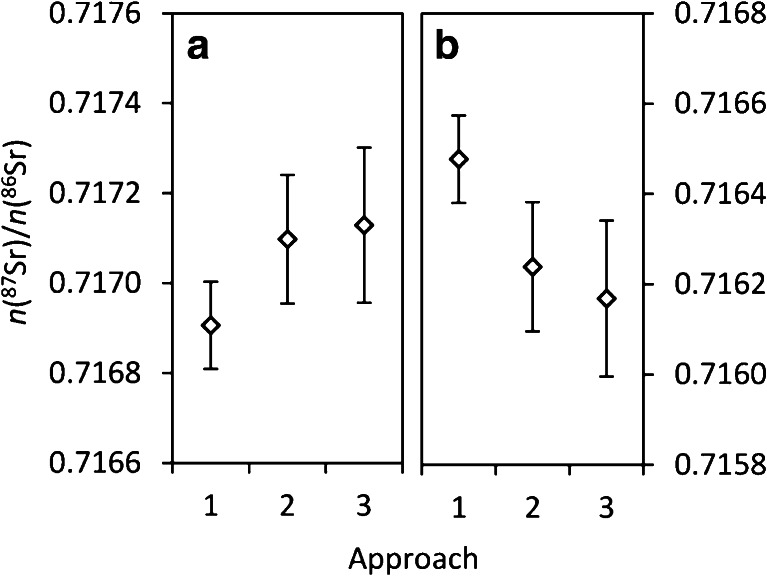
*n*(^87^Sr)/*n*(^86^Sr) of two different wood digest samples evaluated by three different approaches with error bars showing *U* (*k* = 2) without propagation of normalization values and certified values. Parts a and b show different wood samples, which differ in their observed *δ*(^88^Sr/^86^Sr): +0.39 ‰ and –0.60 ‰ for samples a and b, respectively

The values obtained by IIF correction approach 3 usually agree closest to approach 2 with relative differences <0.01 %, whereas approach 1 differs from the others [depending on the observed *n*(^88^Sr)/*n*(^86^Sr) in the sample], sometimes significantly when considering expanded uncertainties according to cases A in Fig. 7. When the shift between *n*(^88^Sr)/*n*(^86^Sr) of the sample versus the standard is negative (negative *δ*(^88^Sr/^86^Sr)_SRM_987_), [*n*(^87^Sr)/*n*(^86^Sr)]_internal_ is higher than [*n*(^87^Sr)/*n*(^86^Sr)]_SSB_ and vice versa. This is demonstrated in Fig. 8 on the example of two different wood samples. Based on Russell’s exponential model, the shift observed for *n*(^87^Sr)/*n*(^86^Sr), which can be attributed to mass-dependent fractionation) is approximately half the shift observed for *n*(^88^Sr)/*n*(^86^Sr) as expressed by the *δ* value. For instance, sample b shows a negative shift by 0.06 % in *n*(^88^Sr)/*n*(^86^Sr) and a shift of 0.03 % between the evaluation approaches 1 and 2. The reason is that the internal normalization contained in approach 1 corrects for MDF (to some extent), whereas the bracketing approaches deliver (ideally) the ratio actually present in the sample, which is defined by both radiogenic and mass-dependent fractionation. A similar observation has been presented earlier for DS TIMS data by Neymark et al. [7], who concluded that the externally normalized *n*(^87^Sr)/*n*(^86^Sr) was not a useful isotope tracer. The MDF reflected in the [*n*(^87^Sr)/*n*(^86^Sr)]_SSB_ may have occurred in nature or during sample preparation or even during measurement as IIF, in case different IIF occurred for the sample and bracketing standards (e.g., due to matrix effects). In combined external and internal correction using Zr (approach 3), different IIF between samples and standards should be corrected for, when both elements respond equally to changed conditions. The lack of significant correlation between Zr and Sr isotope ratios and the close agreement between SSB-Sr and SSB-Zr result values in this study indicate no significant such effect. It remains questionable whether Russell’s model accurately reflects MDF in nature, which the authors of the study themselves doubted [22]. Possible mechanisms of MDF in nature can be divided into equilibrium and kinetic processes and are consequently described by different mathematical relations [97].

**Fig. 9:**
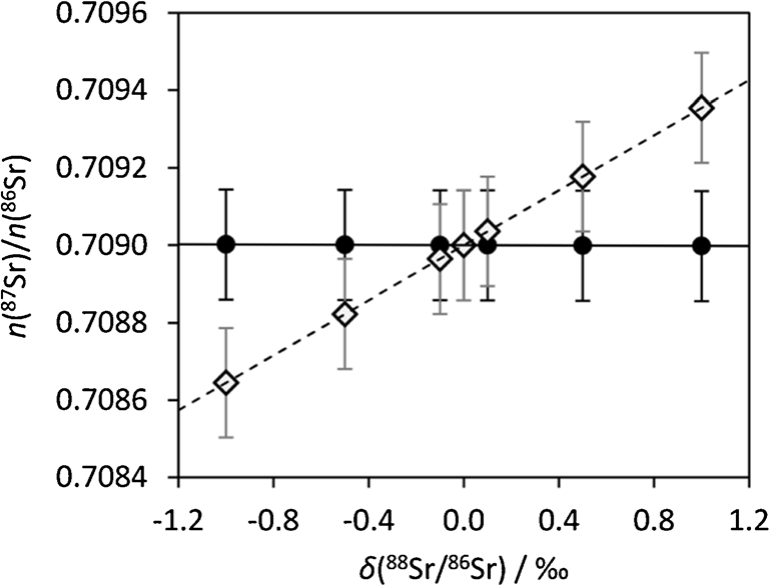
Hypothetical calculation of the effect of MDF in nature [expressed as *δ*(^88^Sr/^86^Sr)] on *n*(^87^Sr)/*n*(^86^Sr) depending on the IIF correction approach; assuming MDF follows Russell’s model. The straight line depicts a *hypothetical* original radiogenic Sr isotope ratio (prior MDF, e.g., the geogenic source of Sr) of 0.7090. After MDF has occurred, the isotopic composition of the sample can be found on the dashed line depending on its new *δ*(^88^Sr/^86^Sr). The black bullets represent the hypothetical result for [*n*(^87^Sr)/*n*(^86^Sr)]_internal_ and the open diamonds show the hypothetical results for [*n*(^87^Sr)/*n*(^86^Sr)]_SSB_ for the same hypothetical samples. Error bars are estimated expanded uncertainties: *U*_rel_ = 0.02 % (*k* = 2)

Consequently, the conditions when the different correction approaches for instrumental isotopic fractionation would give identical result values are: (1) [*n*(^88^Sr)/*n*(^86^Sr)]_sample_ is identical to [*n*(^88^Sr)/*n*(^86^Sr)]_NIST-SRM-987_ (i.e., the certified value 8.37861); (2) no difference in IIF between samples and standards; (3) any deviations from Russell’s model affect standards and samples in the same manner; (4) absence of interferences. Nonetheless, there is evidence refuting statement (1), which implies inexistence of MDF for strontium (e.g., [6]). Figure 9 illustrates, based on theoretical calculations, how MDF would affect the ratio *n*(^88^Sr)/*n*(^86^Sr) and consequently the (ideal) measurement results of approaches 1 and 2 (or 3).

## Conclusions

It is evident that the correction strategy has to be fit for the intended use. In any case, it is most crucial to be transparent regarding all applied calculation procedures, including corrections, normalization, and uncertainty propagation, in order to warrant comparability between different datasets. This goes along with awareness of the information content of the reported result, in case of Sr, with respect to radiogenic variation and (natural) mass-dependent fractionation since different approaches yield different results.

The combined uncertainty calculations have to be accomplished accordingly in order to avoid over- or underestimation of uncertainties. Special attention should be paid to correlations since disregard of correlations generally produces overestimated uncertainties. When uncertainty is propagated according to GUM, including all parameters, the main precondition for lower uncertainties are iCRMs with adequately small uncertainties. Until these are available, measures in the laboratory to minimize uncertainties include optimum tuning for maximum instrument stability and retaining high signal/noise ratios.

Since uncertainties should be fit for the intended use, the relevant input parameters have to be selected carefully. When the main focus is set on relative differences between samples, the propagation of the uncertainty of normalization values (which are the same for all samples in case of the same dataset that is calculated via the same data reduction procedures), is not necessarily required. In these cases, delta values are nonetheless more adequate since they disregard the uncertainty of the anchor value (except for the heterogeneity of the material). When absolute *n*(^87^Sr)/*n*(^86^Sr) ratios are prospective ‘stand-alone results’ for use in databases and, consequently, future transfer to other research questions or comparison to values obtained in different laboratories and/or using different methodology, all uncertainty contributors have to be included.

The results of this paper can be directly transferred to other isotopic systems or considering other methodological approaches. In laser ablation, for instance, where no Sr/matrix separation can be accomplished, the contribution of, e.g., the Rb interference, has to be considered accordingly.

## Electronic supplementary material

ESM 1 (PDF 189 kb)

ESM 2 (XLS 199 kb)
