# Supplementary material for: Evaluation strategies and uncertainty calculation of isotope amount ratios measured by MC ICP-MS on the example of Sr
Source: Anal Bioanal Chem. 2015 Oct 15;408:351–67. doi: 10.1007/s00216-015-9003-9 (PMC4709391; doi:10.1007/s00216-015-9003-9)
Supplement: Supplementary file 1 — (PDF 189 kb) [file 216_2015_9003_MOESM1_ESM.pdf]

Analytical and Bioanalytical Chemistry

Electronic Supplementary Material 1

Evaluation strategies and uncertainty calculation of isotope amount ratios measured by MC ICP-MS on the example of Sr

Monika Horsky, Johanna Irrgeher, Thomas Prohaska

216_2015_9003_MOESM2_ESM.xls

1. Materials and Methods – additional information

Table S1 Sr/matrix separation scheme for extraction chromatography using Sr Resin (Triskem, Bruz, France)

step	reagent	volume / mL
column packing	Sr Resin in 0.3 mol L^{-1} HNO_3	0.5
cleaning	6 mol L^{-1} HNO_3	3
	subb. H_2O	3
	6 mol L^{-1} HCl	3
	subb. H_2O	3
conditioning	8 mol L^{-1} HNO_3	3
sample loading	sample in 8 mol L^{-1} HNO_3	0.5
rinsing	8 mol L^{-1} HNO_3	10
elution	subb. H_2O	2

2. Results and Discussion – additional information

2.1. Blank correction

2.1.1. Faraday cup baselines

Faraday baselines varied by < 0.1 mV (*SD*, *n* = 70) over the course of about 5 years. Short-term Faraday noise measured in 5 s integration intervals was on average 0.016(8) mV (2 *SD*, *n* = 70). Faraday gain calibration factors differed from 1 by less than 1 % and *RSD*s of gain calibration factors of individual cups were between 0.002 % and 0.006 % (*n* = 88 over a period of 1.5 years).

2.1.2. Determination of standard uncertainty and correlation of blank voltages

Different approaches were compared: (1) *SD* of measured signal during a 10 min measurement of an instrumental blank (for standard solutions measured as samples) or procedural blank (for processed wood samples); (2) *SD* of averages of measured signal of several instrumental blanks (for standard solutions measured as samples) or procedural blanks (for processed wood samples). In case the *SD* of the measured signal of one method blank (e.g. a separation blank) is used, three approaches for determining the correlation coefficient *r* and the *SD* were compared: a) determination from the raw measured signals before outlier elimination, b) after elimination of all outliers outside the interval described by the mean ± 2 *SD*, calculated from the original data, and c) from the block averages from 6 blocks (results file, outliers eliminated by the NICE software).

An additional simplified method of blank propagation was tested, which is based on the assumption, that *r* = 1 for the blank signals for Sr. All blanks signals are propagated simultaneously – this means that only one column in the Kragten spreadsheet is required for blank correction contribution, where all three standard uncertainties of the blank signals are added to the respective blank voltage simultaneously.

The calculation of correlation coefficients is sensitive to outliers. Outlier removal generally decreases the signal *SD* for individual blank measurements, while it also reduces the correlation – in case the 'outliers' were caused by an instantaneous increase of Sr in the plasma. This can be caused by e.g. droplets which are released in the nebulizing system or the release of deposited Sr in the introduction system. It is therefore most important to use the same approach for the calculation of *SD*s of blank signals as for the calculation of correlation coefficients. For the correlation of signals within one blank measurement, it makes a difference, whether the correlation coefficient is calculated from raw data (with or without outlier correction) or from the 6 block averages of outlier corrected data. The averaging step removes part of the signal fluctuations and reduces the number of points from 60 to 6 with consequences for the significance of correlations. A one-sided t-test at 0.05 significance level shows, that correlation coefficients < 0.729 cannot be considered significant for *n* = 6. This highlights the disadvantage of calculating the blank *SD* and correlation coefficient from a small number of procedural or instrumental blank average values.

Fig. S1 shows the uncertainty contributors resulting from the different calculation approaches of the blank uncertainties and correlation coefficient. The calculation from one procedural blank (with or without outlier elimination, cases IVa, IVb, Va and Vb) seems most robust, as could be shown by two different standards.

Procedural blanks or method blanks are usually not available in higher numbers ($n \sim 10$), otherwise these would present the optimum alternative to guarantee, that the variability in Sr content and isotopic composition of blanks is accordingly reflected. This approach is, however, complicated by day-to-day differences in instrument sensitivity and between-batch or between-sample differences in Sr contents and accordingly different dilution factors, e.g. between measurement sessions. Case VI in Fig. 4 shows the resulting uncertainty contributor, when no correlations are considered – resulting in overestimation, while case VII shows the commonly applied case, when perfect correlation (r = 1) is assumed, as obtained, when blank signals are propagated simultaneously. In the latter case, the uncertainty contributor is strongly underestimated. We propose to use one measurement of a procedural blank as an estimate for blank uncertainty and for the calculation of reliable correlation coefficients.

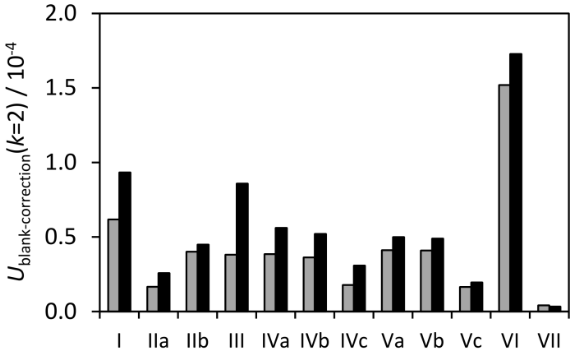

Fig. S1 Combined contribution of blank correction at m/z 86, 87 and 88 to U (k=2) of $n(^{87}Sr)/n(^{86}Sr)$ in a wood digest sample for IIF correction approaches 1 (grey bars), and 2 and 3 (identical, black bars). Roman numerals refer to different blank correction approaches: I using the average signals of 5 instrumental blanks; II *via* the signal of one instrumental blank; III using the average signals of 5 procedural blanks; IV: *via* the signal of one procedural blank processed through digestion and Sr/matrix separation; V: *via* another procedural blank (analogous to V); VI via the same blank as V, but without considering the correlations (r = 0); VII *via* the same blank as V, but assuming perfect correlation of Sr blank signals (r = 1). Small letters refer to the mode of *SD* and r calculation: a from outlier-corrected raw data, b from original raw data, c from blocked data averages.

2.1.3. Monitoring of Kr interference

Monitoring of ^{83}Kr over a period of more than two years gave voltages of (8 ± 60) · 10^{-6} V (2 *SD*, n = 2614) which converts to 4.87(6) mV after baseline correction (assuming constant baseline). This would relate to an estimated ^{86}Kr contribution to the voltage at m/z 86 of 7.31(9) mV. It must be noted, that the total voltage measured at m/z 83 is much lower compared to m/z 86 and consequently more strongly affected by electronic noise, which leads to error magnification. When single data points of several samples over the course of hours are monitored, an approximately sinusoidal (but noisy) curve can be observed with a frequency of around 30 min. The *SD* of individual data points of one sample (60 data points) or over 9 samples (540 data points) are in the same order of magnitude as the *SD* over 2 years. Consequently, our blank correction approach corrects for a mean Kr contribution as well as its variation.

2.2. Correction for interfering ^{87}Rb

Fig. S2 gives the range of residual Rb/Sr (depicted as measured voltage ratio) in 656 digested wood samples after Sr/matrix separation as a histogram. More than 80 % of the samples show total Rb/Sr voltage ratios < 0.015 %.

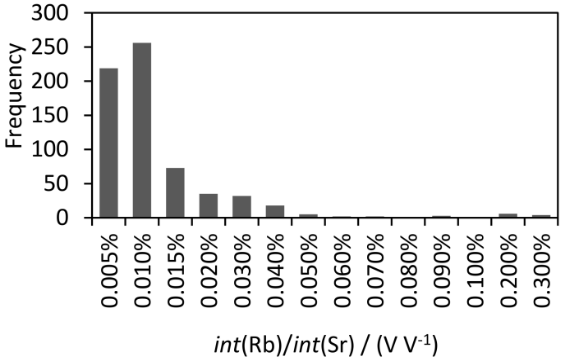

Fig. S2 Histogram of the measured total voltage ratio *int*(Rb)/*int*(Sr) in environmental samples after Sr/matrix separation

2.2.1. *Uncertainty associated to Rb correction*

The experimental data for the standard mixtures show variations with respect to e.g. measurement precision for the different measured ratios. Due to the interlink between them being part of the partial derivatives (sensitivities) determining the individual contributions, conclusions are not trivial from this dataset. Therefore, theoretical calculations based on a fictitious dataset which shows only varying Rb/Sr values and accordingly varying relative precisions of this variable based on an experimentally derived relation ($y = 2.02\cdot10^{-5}\cdot x^{-0.695}$; r^2 = 0.84) were made. They show how the five different contributors to the Rb correction uncertainty contributor relate to increasing Rb/Sr: (1) The blank at m/z = 85 gives a constant absolute contribution independent of Rb/Sr because the partial derivative consists only of constants (and the fractionation factor, which was also assumed constant here). (2) The contributor related to the measurement precision of *int*(^{85}Rb)/*int*(^{86}Sr) increases linearly with the precision, its sensitivity factor is constant. Therefore, it increases proportional to (*int*(Rb)/*int*(Sr))$^{0.305}$. (3) The range of the ratio *n*(^{87}Rb)/*n*(^{85}Rb) in nature, (4) the range of natural *n*(^{88}Sr)/*n*(^{86}Sr) and (5) the measurement precision of *int*(^{88}Sr)/*int*(^{86}Sr) give contributions that increase linearly with Rb/Sr, with slopes decreasing from (3) to (5), *i.e*. decreasing relative importance. Strictly, for (3), the linear increase occurs only in a 'normal range' of Rb/Sr above 0.005 %. All three latter uncertainty contributors do not exceed the contribution of the blank (see point 1) up to a Rb/Sr of 0.5 %. For Rb/Sr > 0.02%, the major contributor to the uncertainty of *n*(^{87}Sr)/*n*(^{86}Sr) related to the Rb correction is the measurement precision of *int*(^{85}Rb)/*int*(^{86}Sr).

2.3. Calibration Strategies: Approach 1

2.3.1. *Internal normalisation n(^{88}Sr)/n(^{86}Sr) values*

The value of *n*(^{88}Sr)/*n*(^{86}Sr) used in internal intra-elemental normalisation has a serious impact on the final resulting [*n*(^{88}Sr)/*n*(^{86}Sr)]$_{internal}$. The value 8.375209 (or its reciprocal 0.1194) relates back to one of the earliest works on Sr isotope ratios by Nier in 1938 [S1] and is based on the analysis of one sample of 99.9% pure Sr metal with compromised traceability to SI units [S2] and an 'uncertainty' estimate of 1%. It was recommended by the IUPAC subcommission on geochronology in 1977 [S3] – and has been routinely used as such since then. That was prior to the publication of the 'Best measurement from a single terrestrial source' in 1982 which it is

still cited in current IUPAC tables [S4]. The above mentioned value is also obtained when dividing the ‘representative isotopic composition’ abundance values given by IUPAC / CIAAW [S4]. Probably, these numbers were derived from the measurement of Nier (as was the standard atomic weight [S5]) or they were obtained by rounding of the ‘Best measurement from a single terrestrial source’ values to 4 decimal places. Division of these latter abundances (rounded to 6 decimal places from a measurement of NIST SRM 987 published by Moore *et al.* in 1982 [S6]) results in a value of 8.37860 (or its reciprocal 0.11935), which is different by 0.04% from Nier’s value. The ‘exact’ numerical value in the stated paper is 8.378612 [S6]. The certificate of NIST SRM 987 gives a value of 8.37861. All values are based on the investigation of two single materials, and therefore do not represent the mean (and dispersion) of several real-world materials. Consequently, it seems more appropriate to use the less rounded value from the certificate. Table S2 lists possible values of $n(^{88}Sr)/n(^{86}Sr)$ for use in internal intra-elemental correction of random (environmental) samples.

Table S2 Compilation of possible values for $n(^{88}Sr)/n(^{86}Sr)$ with pros and cons of their utilisation for internal normalisation

value $n(^{88}Sr)/n(^{86}Sr)$	source	*pro* arguments	*contra* arguments
**8.375209[a]= 1/0.1194**	Nier (1938) [S1]<br>IUPAC [S3]	- comparability to most published data	- NIST SRM 987 certificate not valid<br>- probably does not describe “real world average” even though – if seen as rectangular distribution between 0.1194 +/- 0.00005
**8.378612**	certificate<br>NIST SRM 987;<br>[S6]	- ideally suited for NIST SRM 987<br>- provided with uncertainty<br>- use of one value for all samples	- probably does not describe “real world average”<br>- limited comparability as most studies normalise to 1/0.1194<br>- which uncertainty to use, when applied to real world samples?
**estimate for AVG ‘real world’, e.g. bulk silicate earth**	literature compilation;<br>e.g. [S7]	- certain samples are well described (silicate rocks)	- many samples may differ substantially from bulk silicate earth<br>- best estimate might change with time and require revision; causing impaired comparability
**different values for different samples**	literature; own measurements	- maybe advantageous for certain applications (e.g. tracing across different matrices)	- compromised comparability between datasets/studies

*It has to be noted, that even though the original publication provides a value (0.1194) with 4 significant digits, the reciprocal value (8.375209) is used with 7 significant digits throughout the literature.

2.3.2. Literature Review about reported $\delta(^{88}Sr/^{86}Sr)$

A histogram was created from a compilation of values for $\delta(^{88}Sr/^{86}Sr)$ as stated in publications: It includes data generated by DS TIMS [S8-12], SSB MC ICP-MS [S13-16], MC ICP-MS using Zr as internal standard [S17,18], or a combination of SSB and Zr internal normalisation [S7,19], and DS MC ICP-MS [S20] (Fig. S3). All available data (n = 337) of natural samples or reference materials (except results for NIST SRM 987) were used. Duplicate determination of a certain (reference) material by different authors was not eliminated: for example, 6 results for IAPSO seawater standard are included. Due to the high total number of samples in a similar range, these duplicates should not significantly bias the histogram. It should be noted, that only a small number of biogenic samples (plant, animal tissue) is included in the dataset, while the majority consists of geological material (water, rocks, soil). 75% of the reported values lie between 0.1 and 0.4 ‰. When maintaining the value 0 ‰ as normalisation value and assigning a standard uncertainty (SD) of 0.25 ‰ for describing ‘typical terrestrial material’, 94% of the present dataset are included in the range with $k = 2$. It seems more appropriate to

propagate this uncertainty instead of the uncertainty on the certificate, when normalisation is applied to real-world materials.

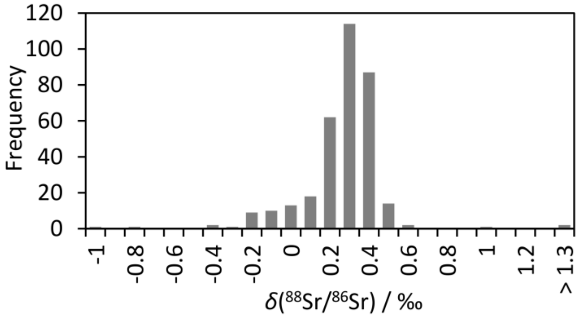

Fig. S3 Histogram created from available $\delta(^{88}Sr/^{86}Sr)$ data from literature [S13,14,8,7,9,17-19,15,10,20,11,16,12]

In general, care must be taken, when values generated by different methods are compared. While certain methods, such as DS TIMS deliver absolute isotope amount ratios, others (e.g. SSB MC ICP-MS) measure delta values directly. The conversion from one data type to the other always requires a best estimate of the true value for the CRM (and the knowledge and propagation of an associated uncertainty). It is clearly crucial, that the used conversion values are clearly stated along with further normalisation procedures that are typically applied to 'adjust for unknowns' – *i.e.* bias. Values of $n(^{88}Sr)/n(^{86}Sr)$ obtained by MC ICP-MS must be critically assessed as there is increasing evidence, that high Ba concentrations compromise accurate $\delta(^{88}Sr/^{86}Sr)$ determination [S13,21]. Additionally, interfering ^{86}Kr must be corrected for [S13,7].

2.4. Calibration Strategies: Approach 3

The dependence of the expanded uncertainty contributor of IIF to U ($[n(^{87}Sr)/n(^{86}Sr)]_{SSB\text{-}Zr}$; k=2) on the *RSD* of the Zr voltage ratio in the sample is shown for a sequence of wood digest samples in Fig. S4.

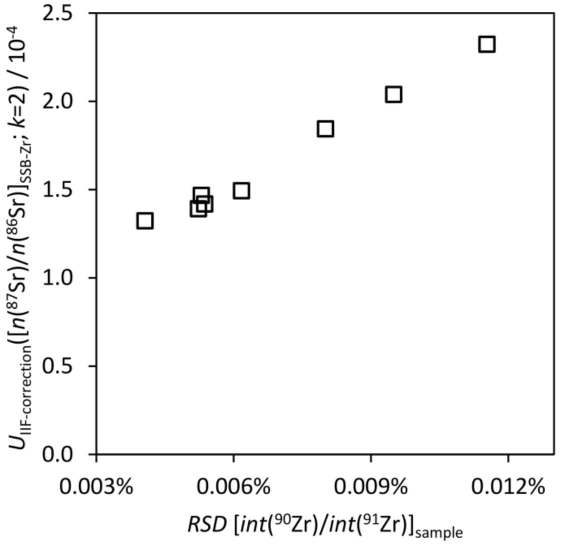

Fig. S4 Contributor related to IIF correction approach 3 to U (k=2) of $n(^{87}Sr)/n(^{86}Sr)$ *vs. RSD* of the measured ratio $int(^{90}Zr)/int(^{91}Zr)$ in wood digest samples

3. References

S1. Nier AO (1938) Physical Review 54(4):275-278

S2. Brach-Papa C, Van Bocxstaele M, Ponzevera E, Quétel CR (2009) Spectrochim Acta, Part B 64(3):229-234

S3. Steiger RH, Jäger E (1977) Earth Planet Sci Lett 36(3):359-362

S4. Berglund M, Wieser ME (2011) Pure Appl Chem 83(2):397-410

S5. De Laeter JR, Böhlke JK, De Bièvre P, Hidaka H, Peiser HS, Rosman KJR, Taylor PDP (2003) Pure Appl Chem 75(6):683-800

S6. Moore LJ, Murphy TJ, Barnes IL, Paulsen PJ (1982) Journal of Research of the National Bureau of Standards (United States) 87(1):1-8

S7. Moynier F, Agranier A, Hezel DC, Bouvier A (2010) Earth Planet Sci Lett 300(3-4):359-366

S8. Krabbenhöft A, Eisenhauer A, Böhm F, Vollstaedt H, Fietzke J, Liebetrau V, Augustin N, Peucker-Ehrenbrink B, Müller MN, Horn C, Hansen BT, Nolte N, Wallmann K (2010) Geochim Cosmochim Acta 74(14):4097-4109

S9. Böhm F, Eisenhauer A, Tang JW, Dietzel M, Krabbenhöft A, Kisakurek B, Horn C (2012) Geochim Cosmochim Acta 93:300-314

S10. Raddatz J, Liebetrau V, Rüggeberg A, Hathorne E, Krabbenhöft A, Eisenhauer A, Böhm F, Vollstaedt H, Fietzke J, López Correa M, Freiwald A, Dullo WC (2013) Chem Geol 352:143-152

S11. Neymark LA, Premo WR, Mel'nikov NN, Emsbo P (2014) J Anal At Spectrom 29(1):65-75

S12. Pearce CR, Parkinson IJ, Gaillardet J, Charlier BLA, Mokadem F, Burton KW (2015) Geochim Cosmochim Acta 157:125-146

S13. de Souza GF, Reynolds BC, Kiczka M, Bourdon B (2010) Geochim Cosmochim Acta 74(9):2596-2614

S14. Knudson KJ, Williams HM, Buikstra JE, Tomczak PD, Gordon GW, Anbar AD (2010) Journal of Archaeological Science 37(9):2352-2364

S15. Ma J, Wei G, Liu Y, Ren Z, Xu Y, Yang Y (2013) Chin Sci Bull 58(25):3111-3118

S16. Vollstaedt H, Eisenhauer A, Wallmann K, Böhm F, Fietzke J, Liebetrau V, Krabbenhöft A, Farkaš J, Tomašových A, Raddatz J, Veizer J (2014) Geochim Cosmochim Acta 128:249-265

S17. Charlier BLA, Nowell GM, Parkinson IJ, Kelley SP, Pearson DG, Burton KW (2012) Earth Planet Sci Lett 329-330:31-40

S18. Liu HC, You CF, Huang KF, Chung CH (2012) Talanta 88:338-344

S19. Irrgeher J, Prohaska T, Sturgeon RE, Mester Z, Yang L (2013) Analytical Methods 5(7):1687-1694

S20. Shalev N, Segal I, Lazar B, Gavrieli I, Fietzke J, Eisenhauer A, Halicz L (2013) J Anal At Spectrom. 28(6):940-944

S21. Scher HD, Griffith EM, Buckley WP (2014) Geochem Geophys Geosyst 15(2):499-508
